# Implementing the ILAE 2025 seizure taxonomy in medical software

**DOI:** 10.3389/fncom.2026.1877135

**Published:** 2026-10-13

**Authors:** Andrew Knight, Sanna Jousea, Jukka Peltola

**Affiliations:** 1 Faculty of Medicine and Health Technology, Tampere University, Tampere, Finland; 2 Neuro Event Labs, Tampere, Finland; 3 Department of Neurology, Tampere University Hospital, Tampere, Finland

**Keywords:** bitfield, epilepsy informatics, ILAE 2025, interoperability, medical software, seizure classification, seizure descriptors, TypeScript

## Abstract

The International League Against Epilepsy (ILAE), working via a multidisciplinary task force, has issued a position paper with updated seizure classification guidelines (2025) replacing its previous guidance (2017), establishing a revised de-facto standard for clinical assessment and classification of epileptic seizures. The 2025 model simplifies the system, making it easier to adopt in different clinical settings while also clarifying the taxonomic rules, the definition of consciousness, and the application of phenomena-based descriptors. As a result, clinical practitioners, technologists, and medical software developers also face the challenge of adopting the new standard in their applications and workflows. To aid in the realization of this update in medical device software, this article presents a proposed numerical structure for ILAE 2025 edition of the seizure classification system as a definition of interacting bitfields, establishing an open reference implementation to support common storage and interoperability between software environments. Assigning each orthogonal concept to a single bit, we encode the core ontology and expanded descriptor set so that they fit into 32-bit and 128-bit unsigned integers, respectively. TypeScript, known for its expressiveness and practical applicability, is used as the implementation language for these definitions. Examples are provided to help implementers incorporate the proposed specification into their software, as well guidance for mapping from the ILAE 2017 type system to the updated ILAE 2025 type system. The reference specification, with test cases and examples, is maintained in a public code repository and available under a permissive license.

## 1 Introduction

The recent position paper from the International League Against Epilepsy (ILAE) (Beniczky et al., 2025a) calls for clinicians to adopt a modernized, streamlined approach to the classification system last updated in 2017 (Fisher et al., 2017b). The 2025 edition simplifies the system, making it easier to adopt in different clinical settings while also clarifying the taxonomic rules, definition of consciousness, and application of phenomena-based descriptors. To aid clinical deployment of the new taxonomy, a practical guide (Beniczky et al., 2025b) was published later that year—in much the same manner as the 2017 instruction manual (Fisher et al., 2017a)—offering specific guidance on the application of consciousness determination and descriptors. While this update does not represent a formal specification from a standards organization, it does represent something approaching that: a detailed, clinically-applicable consensus procured by a panel of community thought leaders. Accordingly, it also calls for information technology providers to adopt the new de-facto standard, as medical software and devices must incorporate the current state of the art and adhere to the current standard of care within clinical practice. Concurrently, technologists must consider interoperability, maintainability, and extensibility within the chosen solution.

Given the rise in use of artificial intelligence (AI) in epilepsy, updating the labeling standard is perhaps more important than ever. As a recent expert epilepsy working group (the European Reference Network for Rare and Complex Epilepsies) (Tchaicha et al., 2026) frames it, “*large language models (LLMs) have the capacity to extract and interpret unstructured data from EMRs, which is one of their strengths […] [h]owever, further validation and clinical implementation are needed to ensure accuracy, reliability, and integration into routine practice*”. As part of addressing these challenges, the authors envisage their research network to be “*uniquely positioned to create large, high-quality datasets […] compiled by expert humans, [that] can serve as a foundation for training AI algorithms and for implementing and testing advanced technologies through clinical trials*”. Clinical networks, interoperable software systems, and an updated seizure taxonomy present a crossroads of opportunity: unlike unstructured text, the ILAE type system implies a set of fixed numerical values, tailored to be used by expert humans for the curation of existing and future medical databases in the documentation of seizure classifications and recorded epileptic events. It also presents an opening to leverage human-in-the-loop methods for ensuring the quality of labels generated by AI models.

As practitioners and technologists working in the epilepsy space, we require a tangible, conformant implementation for the software we use and build and within the connected systems with which it interacts. To this end, we propose a compact representation of the updated classification system based on binary flags and partitioned numeric bit range fields, such that the core ontology can be represented in a 32-bit unsigned integer. We further define flags for use of descriptors, allowing for the expanded classification model to reuse the numeric definitions of this base ontology, and show that it is possible to fit the entire predefined semiology into 128 bits. Through this bitwise approach, we demonstrate how these flags can be used to identify different characteristics of specific classifications with simple bitwise arithmetic, encouraging elegant and performant representations in the underlying software. Additionally, we discuss how the previous 2017 model maps to the 2025 model, and provide examples and test cases for adherence to demonstrate compliance with clinical expectations. Underpinning the academic value of this endeavor is a more practical goal: in our continued work within video-based seizure detection (Rai, 2024), we are mapping our existing ILAE 2017 labels to the 2025 standard.

The code associated with this proposal has been implemented in TypeScript and serves as a reference implementation; it is available in a public repository (Knight and Neuro Event Labs, 2026) and provided under a permissive license (MIT). As the implementation is nascent, and has not been endorsed by the ILAE or any other recognized standards organization, it should be considered exploratory and experimental at the time of this writing. Our intent in releasing this proposal is to foster discussion within the community and encourage research into this space, with the eventual outcome of establishing an open consensus standard adopted by a range of software solutions.

## 2 Method

The primary scope of this work is to define a representation of the ILAE 2025 taxonomy that fits into standard numeric types across most general programming contexts, so that it can be used by language runtimes as well as relational databases while retaining portability and minimizing differences in numeric operations between environments. Ideally, such a representation supports direct use via bitwise operators, as this offers a familiar and computationally efficient method of querying stored information. Certainly, it is common for runtimes to support bitwise operations on integer types natively, but any structure that supports bitwise storage and functionality might be considered a bitmap. For the sake of clarity, the concepts below apply to the runtime representation of this binary encoding, particularly when bitwise operations are used. When specifically addressing related concepts such as serialization, database storage, and user interface choices, those aspects are highlighted.

### 2.1 On the use of binary arithmetic

While a familiar concept to those doing low-level programming work, the concept of binary representation may not be second nature to most of those working in the epilepsy domain. Through the use of binary arithmetic, it is possible to encode every possible value of the type system (e.g., every possible seizure type) as a combination of its constituent features, while also implicitly encoding relationships between features common to two or more values. Many features will simply be binary: that is, either on or off; true or false, also known as a boolean value. These values can then be compared using boolean logic, and by extension, the binary operators. This allows us to model a given phenomenon as a flag, meaning a single bit that is contained in every value that shares that feature, and is not present in any value that does not.

It is especially useful to express numbers in binary notation when each place value has been assigned an “on or off” meaning. As a convenience for those readers unfamiliar with the core concepts, we provide the following refresher to aid in reading the remainder of the article:

1. Numbers may be expressed in binary notation as a series of 1s and 0s, and can also be expressed with a different *radix*, or base, such as the commonly-used base-10 (decimal) or base-16 (hexadecimal) options.
2. Each place value in a binary number corresponds to one bit (1 or 0) and represents decimal 2^N^ (where N is its 0-indexed position), with the total value of the number being the sum of these place values (conversely, each value's bit position can be calculated from its base 2 logarithm).
3. Numbers can be combined using binary operators such as OR (denoted as a pipe, |) or compared using AND (denoted as an ampersand, &) to derive a new number where each bit in the first operand has been combined/compared with its corresponding bit in the second operand. ORed values “inherit” bits from both operands (“parents”), while ANDed values reveal whether the first operand's bits are present in (“inherited” by) the second operand's.
4. Numbers can be shifted (left or right) by any number of places, resulting in new numbers that retain the original binary pattern, but in a new position (with the caveat that bits shifted right past 0 are lost).
5. A number (whether a single bit or the entire value) is considered “truthy” when it is non-zero; zero is considered to be “falsy”.

Applying these rules to a core example in epilepsy, consider encoding the concept of “epileptic” as a binary value: is it epileptic or not? This is a quintessential research question in epilepsy diagnosis, particularly when the evidence is based on patient or caregiver verbal description. Even when a video recording of the event is available, a video electroencephalogram (EEG) produced by an epilepsy monitoring unit (EMU) remains the gold standard for clinical diagnosis of epilepsy.

However, clinical observers will quickly point out that a third option exists: unknown. Like any evidence-based medicine, epilepsy diagnosis involves multiple inputs to increase the certainty given the totality of the information. We may simply not have enough information at a given point to determine whether the event is epileptic or nonepileptic, and must label it as unknown. Such a “tri-boolean” would then require 2 bits, and might correspond to a representation such as:

- binary 00 (decimal 0): unknown
- binary 01 (decimal 1): epileptic
- binary 10 (decimal 2): nonepileptic
- binary 11 (decimal 3): ?

In this representation, 0 carries no value information apart from the literal meaning of carrying no information (“unknown”). The value 1 indicates that the event is epileptic, and the value 2 indicates that it is nonepileptic. Note for the purposes of this feature, we need not assume that any value indicates a seizure: only that it is unknown, epileptic, or nonepileptic in nature.

While this appears to be a reasonable solution for a 2-bit system, it leaves one value unassigned (binary 11, decimal 3): what should this value represent? When treated as a bitwise mask, it can be used to perform a number of potential search functions:

**Figure:**
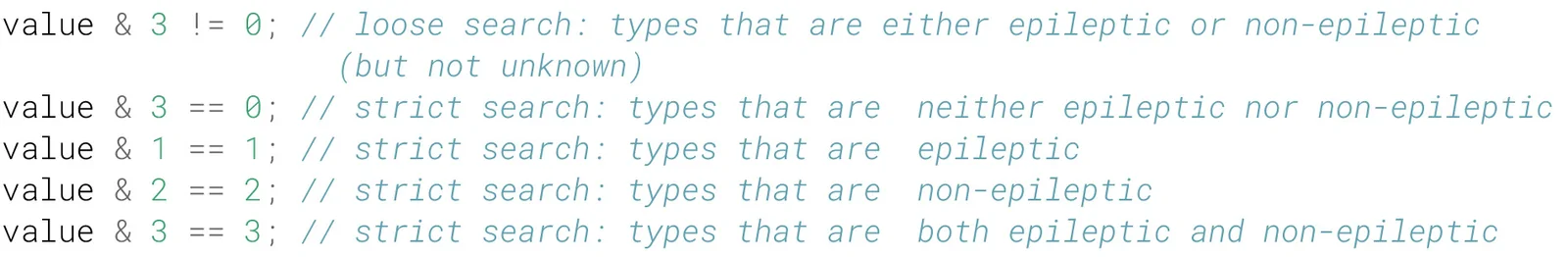


**Note:** The code above and all snippets in this article are intended to be syntactically-valid TypeScript, but may not be directly meaningful in a program due to missing references or typesetting artifacts. They should be considered illustrative examples; the reader should refer to the code repository for usable software.

In a database of medical events, the first four cases might all be reasonable searches. The last case is more interesting, because the value 11 (decimal 3) represents a type that is not likely to exist in the standard ontology (meaning an event that is classified as both epileptic and nonepileptic). By using two bits, we've effectively treated these two traits as separate – but not mutually exclusive – despite the clinical assumption that they must be. Therefore, this becomes a model example of how the type system must partition the bitfields in a way that allows us to avoid erroneous logical conclusions about the phenomenon represented by the type. In the following sections, this principle will be applied repeatedly to complete a working numeric ontology for the updated seizure classification system.

### 2.2 Implementation constraints

A common aspect of interoperability is to define an abstraction level that is equivalent across environments. As pointed out earlier, a bitfield can be effectively implemented as an (unsigned) integer in most programming languages. Native support for unsigned integers of various widths (i.e., byte sizes or bit depths) exists in popular languages such as C, C++, Rust, and Java. JavaScript, arguably the most consequential programming language for modern applications, offers a simplified model, treating numbers (via the Number type) as IEEE-754 double-precision floating point values (equivalent to the double type in C/C++/Java and f64 in Rust). Python 3 (The Python Software Foundation, 2026) is somewhat similar in that it provides an abstraction over all numeric types, but has various extensions for implementing integers of arbitrary size. When working with the native Number type in JavaScript, bitwise operations treat (or internally convert) the operands to 32-bit integers, creating a practical limit to the number of flags available: 32, with some special handling required to use the highest (sign) bit without causing representation errors. This is in contrast to languages such as Rust (or C++, using language extensions) that offer native integers of up to 128 bits, or any language that supports an arbitrary precision library. Beyond that, many languages have the capability to define bitmaps of arbitrary size using more conventional class-based methods (consider the C++ standard library's space-efficient specialization of std::vector<bool> as a partial example).

Modern JavaScript runtimes offer the BigInt (for “big integer”) API, providing bitwise operations for values of an arbitrary number of bits, but also requiring explicit handling. Using big integer features would then imply that all other languages would need an equivalent solution. In effect, JavaScript may be viewed as the most constrained of these languages; the lowest common denominator: if a language feature is used in this specification, it must be implementable in JavaScript, with the presumption that such possibilities are available in other languages. Therefore, the following specifications have been constrained to keep the core features within 32 bits, with arbitrary bit widths (via a big integer or similar implementation) for the expanded features.

### 2.3 TypeScript as an implementation language

As JavaScript is a lexically untyped language (meaning that types are not explicitly stated, unlike in C or C++), TypeScript, described as “*JavaScript with syntax for types*” (Microsoft, 2026), is used as a *transpiler* to author code that is type-checked at build time (but still operates as JavaScript at runtime, allowing to run in web browsers and other environments such as Node.js (OpenJS Foundation, 2026b). For the purposes of creating an interoperable specification that assumes JavaScript as the baseline feature set, this makes TypeScript an excellent fit for defining and describing the numeric model. Unlike plain JavaScript, TypeScript's build-time type checks include features that aid in numeric modeling, and these are exposed to the developer via the integrated development environment (IDE). One of the most useful features is that enumerated subsets can be defined as types, allowing the values to “inherit” the enumerated type while being constrained to a value. For example, one can declare a pair of constrained numeric types simply by listing all the allowed values:

**Figure:**
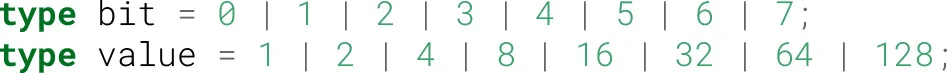


These are similar to enumerated values, and in fact could be declared as an enum instead. In this example, bit represents any value from 0-7 (but nothing else), and value represents all the corresponding decimal values of a number with only the respective bit set (i.e., when 1 is left-shifted by the bit value). In other words, these types can be used to represent the zero-indexed bit positions and corresponding values of an 8-bit unsigned integer. Using these, an object could be defined to provide all the corresponding numeric values for the individual bits, and the transpiler ensures that only (and all) keys of type bit are present, and all are of type value:

**Figure:**
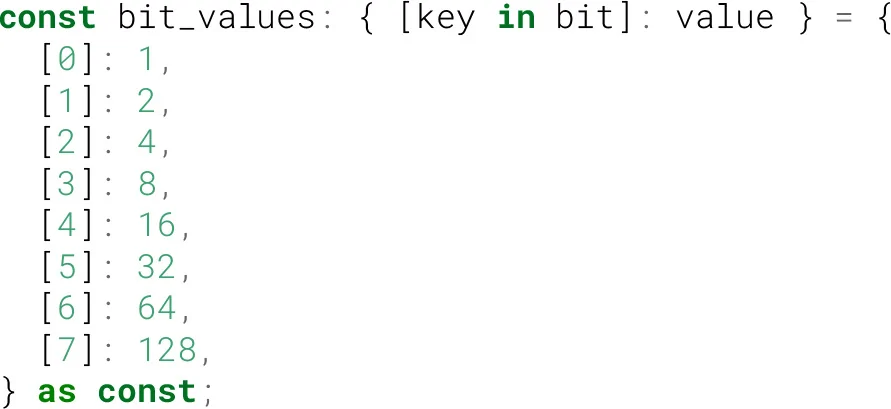


More generally, any interface accepting a bit or value would be statically (compile-time) checked to allow only the listed values (TypeScript users may note that the equivalent of this feature is also available under the templated Record utility type). We can further define derived types, such as bit value ranges (i.e., masks), using the following approach:

**Figure:**
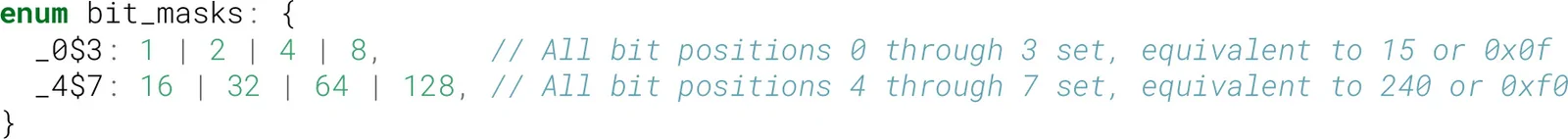


This effectively gives self-describing names for well-known numbers, without having to remember or compute the numbers themselves (this becomes increasingly important as the numbers grow large and unmemorable). The usefulness of these language features in the context of the ILAE 2025 type system becomes more apparent as we consider the following implementation proposal.

### 2.4 Proposed structure

Returning to the original example (epileptic vs. non-epileptic) and considering the language constraints described thus far, we can begin to define a seizure type enumeration in TypeScript. To avoid having to compute powers of two manually, we define a helper enum, Bit:

**Figure:**
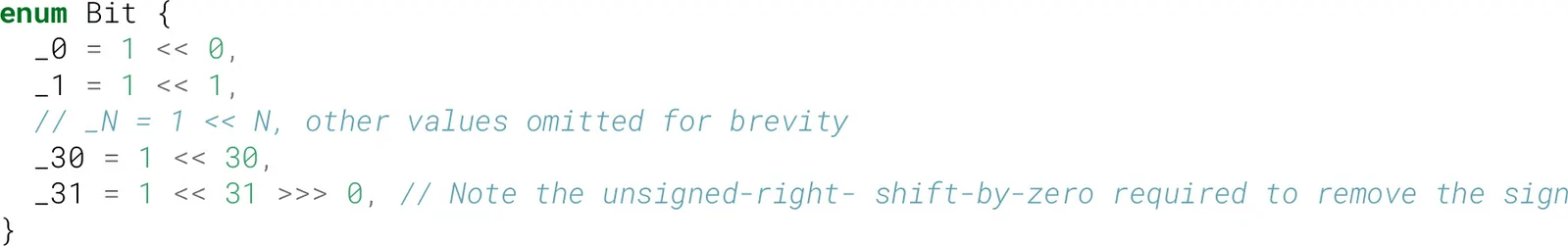


…and then define the first few bits:

**Figure:**
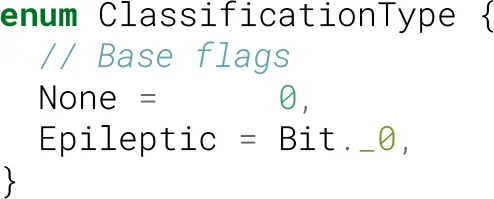


We start with a None value of 0, conveying a meaning of both “no information” and “unknown”. While our original example defined a third value, nonepileptic, it is not immediately required for modeling the ILAE 2025 type system—in fact, all types in this system are implicitly epileptic, suggesting that no epileptic-signaling bits are needed at all. However, given the goal of integrating this numbering system into medical device software that deals with indications outside of epilepsy, we propose assigning a designated bit to this pivotal attribute.

It is worth noting that, under JavaScript runtimes, “bit 31” requires special handling to ensure that it is treated as an unsigned integer. Otherwise, including this value may lead to unexpected introduction of negative signed integer values into the encoding. Therefore, any predefined values utilizing this bit should include the idiom “unsigned right-shift zero” to ensure that its stored value is treated as unsigned. By incorporating this idiom into the library, the pitfall can be mitigated.

To help clarify structural aspects to follow, the core concepts have been defined in Table 1 (as they apply to the proposed methodology).

**Table 1:** ILAE 2025 taxonomy teminology with respect to the proposed encoding scheme.

Concept	Description
Bit	A single true/false (1/0) value embedded within a numeric type
Trait	A specific characteristic of a seizure, seizure type, or event, ideally encodable in a single bit
Mask	A group or combination of numeric values (bits) used to test for specific traits or groups of traits
Classification	The determined type of a seizure (following the ILAE 2025 methodology)
Descriptor	An optional characteristic (trait) applied to describe a seizure beyond its base classification
Canonical	Referring to a specific classification or descriptor explicitly documented in the ILAE 2025 position paper
Extension	An encodable concept (e.g., a new trait or combination of traits) not defined by the canonical lists

#### 2.4.1 Seizure origin

In ILAE 2025, the primary defining feature of the seizure is its “origin”: one of Focal, Generalized, Unknown, Unclassified. The origin, or class, replaces the more overloaded term “onset” (or “focus”) used by ILAE 2017, as the paper's authors note that onset alone cannot be used to fully distinguish between focal and generalized seizures. The article states that focal seizures “*are defined as originating within networks limited to one hemisphere*” while generalized seizures “*are defined as originating at some point within, and rapidly engaging, bilaterally distributed networks*”. Accordingly, the differentiation between focal and generalized seizures requires knowledge of the electrographic activity within the brain (typically via EEG recordings) or through semiological features that infer lateralization and/or localization of the epileptogenic zone. If there is insufficient evidence to classify the seizure as focal or generalized, the Unknown (whether focal or generalized) label applies. However, the authors provide an additional “escape hatch” with the Unclassified class: to be used as a “temporary class, when no further information is available about the seizure” in “cases where there is no available information to characterize the seizure, but the clinician is confident that the event is an epileptic seizure”.

Accordingly, all four main classes are indeed Epileptic, but the “unclassified origin” concept indicates an information gap not present in the other classes. However, we also must acknowledge that the “unclassified” is not anticipated to be used the same way as any of the other classes; it is, in effect, equivalent to UnknownOrigin but with the temporary “flag of uncertainty” until the class is resolved. Therefore, we propose not defining an UnknownOrigin value, but rather introducing an “otherness” concept (placed in the final bit, as Other) to be used when a generic extension mechanism is required. The UnclassifiedSeizure type then includes this Other bit.

**Figure:**
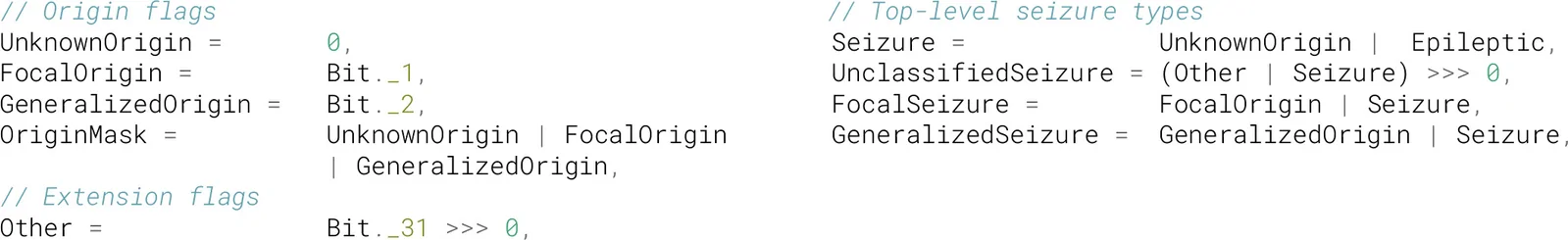


This model has the advantage that all top-level seizure types now inherit the value Seizure (meaning a seizure of unknown whether focal or generalized). It complicates the origin-based search slightly, as implementers must also either exclude the final bit during search (a useful approach for excluding extensions, as we'll discuss below) or otherwise handle the entire returned value. We find this to be a suitable trade-off for this use case, as we expect the use of the UnclassifiedSeizure to be a temporary state in most clinical workflows.

The astute reader will also note that combining UnknownOrigin with another value has no numeric effect, because it is zero. However, it has two advantages: it documents the relationship between concepts, and allows for minimal code changes should the concept later be changed and assigned a bit. Such design should only be used for implicitly unknown or default states, though, as such an encoding can not simultaneously represent “undefined” concepts like “unset” or “not recorded”: in the presence of default states, these “undefined” states should be tracked separately. Moreover, such zero-value types may lead to semantic search failures when they are erroneously treated as normal valued bits. The implementer cannot search directly for UnknownOrigin by only testing for the presence of the (non-existent) bit; it will require that the match is done on a set of bits (in this case, OriginMask) and compare the result with zero (i.e, UnknownOrigin). As this works in the non-zero case as well, implementers can implement these “strict” comparisons for most searches (even when only considering a single bit). Perhaps more robustly, a search function could enforce the use of a non-zero mask when searching for a zero-value result.

Functionally, one-bit matches for FocalOrigin or GeneralizedOrigin would yield only those labels within those respective classes. A ClassMask (two-bit) search compared to a specific bit value (e.g., UnknownOrigin, FocalOrigin, or GeneralizedOrigin) would do the same, while a loose/truthy match would return only types of focal or generalized origin. Strict searches for combined flags like Focal | Generalized would only return results if a seizure were labeled both focal and generalized; something that should not be possible within the ILAE 2025 model. Therefore, it is important for implementers to understand both the semantics of the search and the underlying data; they may wish to implement type constraints (such as an enumeration check) to prevent the storage of semantically-invalid values.

#### 2.4.2 Focal and unknown origin seizure subtypes

For the Unknown and Focal classes, the next step in classification procedure is to identify the patient's consciousness during the seizure, an assessment of requiring clinical testing of the patient's ictal awareness and responsiveness. Consciousness can be modeled similarly to the origin:

**Figure:**
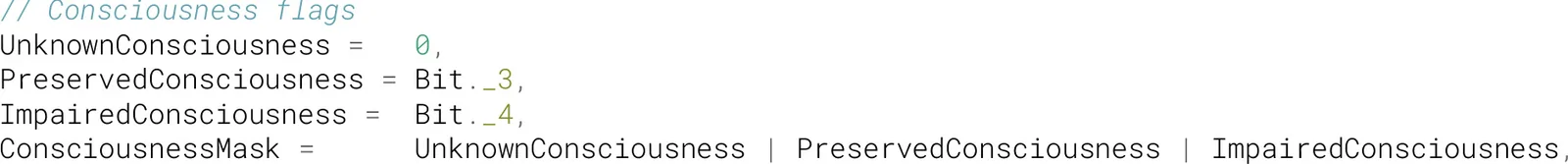


As unknown consciousness is the default state (analogous to the UnknownOrigin trait), there is no need to assign it a truthy value. Similarly, the combination PreservedConsciousness | ImpairedConsciousness likely has no clinically meaningful value on its own, although it is useful for searching for values that have their consciousness known.

#### 2.4.3 Generalized origin seizure subtypes

For generalized seizures, the next rule is concerned with assigning the behavioral subtype. Users of the ILAE 2017 may consider this the most significant change in ILAE 2025: there is no longer a distinction between non-motor and motor seizures, and (with the exception of tonic-clonic seizures) the non-generalized classes do not have specialized behavioral subtypes. This reasoning is rooted in the better clinical understanding of generalized epilepsy syndromes, where behaviors are more consistent across patients. As a numerical consequence, we can list the various observable features that are present in these seizures:

**Figure:**
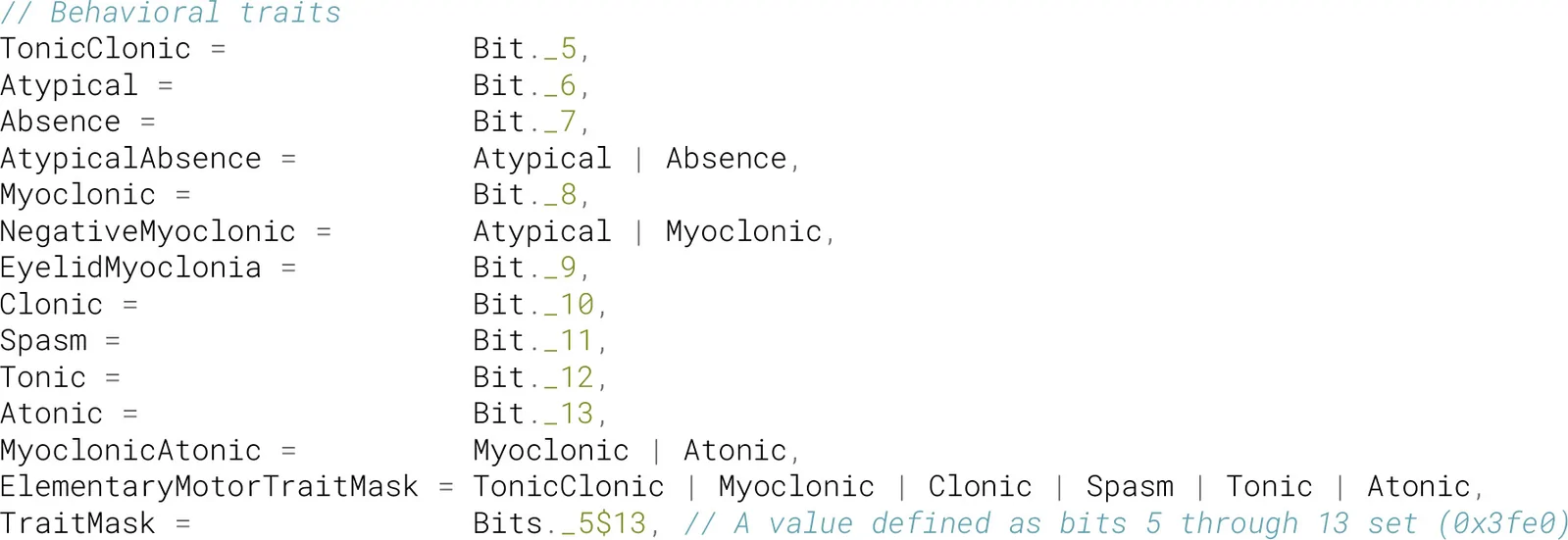


Some liberties were taken to establish a compact structure that balanced the need to support realistic clinical use cases with accurate separation of the underlying orthogonal clinical concepts. One of the core questions is whether a given flag represents an “is a” or a “has a” relationship. For the purposes of this proposed ontology, we consider the behavior flags to designate an “is a” relationship when defining the seizure type. For example, the TonicClonic value was chosen as a single bit rather than a combination of Tonic and Clonic values. While tonic-clonic seizures always present with bilateral tonic-clonic activity, presentation can be variable (Beniczky et al., 2022). While the term tonic-clonic suggests both tonic and clonic elements, the tonic-clonic phenomenon is distinct. Accordingly, a tonic-clonic seizure does not “inherit” or “extend” a tonic or clonic seizure: all three are independent behaviors. By separating them, a number of ambiguities can be avoided; for example, Tonic and Clonic seizures are now always considered generalized seizure classifications in ILAE 2025, but can also describe tonic and/or clonic manifestations in focal or unknown origin seizures. In this way, certain semiological signs associated with a given seizure type, (such as figure-of-four in tonic-clonic seizures) can be used to describe the observable phenomena irrespective of the seizure's basic classification.

Unlike the tonic-clonic case, the absence behaviors (typical, atypical) and myoclonic behaviors (negative myoclonic, myoclonic-atonic) have been implemented using combinators to reflect the overarching subtype. For the absence seizures, the practical guide is clear that absence seizures are either typical or atypical, allowing the “atypical” feature to be defined on its own rather than defining two subtypes. For the various myoclonic subtypes, it is observed that they present in a number of closely related syndromes, making it a reasonable conclusion to have each of them inherit the Myoclonic flag. This is supported by the literature, as myoclonic seizure types tend to be present in a number of related syndromes (Riva et al., 2024).

Notably, we propose to differentiate between GeneralizedMyoclonicSeizure and GeneralizedNegativeMyoclonicSeizure by including the Atypical bit (also used when defining the AtypicalAbsenceSeizure type). In this manner, the two types are differentiated while still inheriting the myoclonic value, analogous to the way that absence seizures are handled. This is furthermore consistent with the position paper, as well as the 2017 response to the concerns around negative myoclonus raised by the SCORE authors: “*[d]espite these diagnostic issues, it would be acceptable to indicate that myoclonic seizures can have either positive or negative manifestations*”. This choice is made due to the scarcity of bits in the base classification (restricted to the 32-bit integer) compared with the observed benefit of defining a common “atypical” trait. Accordingly, single-bit queries for Atypical will yield both AtypicalAbsence and NegativeMyoclonic seizures (a perhaps unexpected outcome), but strict multi-bit queries for either type will not. Furthermore, the ostensibly more common searches for Absence or Myoclonic bits will not yield any unintended values from their respective seizure groups, but will still capture the “atypical” subtypes. This problem is not unique to the Atypical bit though: as noted above, tonic and clonic features can appear in focal seizures, despite only generalized seizures reflecting those canonical types in ILAE 2025 (as opposed to ILAE 2017, which defined both tonic and clonic focal seizures). As the nuance of this design may sometimes violate the principle of least surprise, its consequences are further explored below.

When considering consciousness, the practical guide states that “if consciousness is impaired at any point during the seizure, even if it is preserved at the onset, the seizure is classified as having impaired consciousness”. It goes further for certain seizure types, stating that “*by definition, both absence seizures and generalized tonic-clonic seizures involve impaired consciousness*”, and that “*consciousness is impaired during [focal-to-bilateral tonic-clonic] seizures*”. Therefore, we propose including the ImpairedConsciousness flag in all tonic-clonic and absence seizures.

#### 2.4.4 Resulting canonical types

By combining all the definitions above, the remaining (in addition to the four values above) “canonical” seizure list can be defined as follows:

**Figure:**
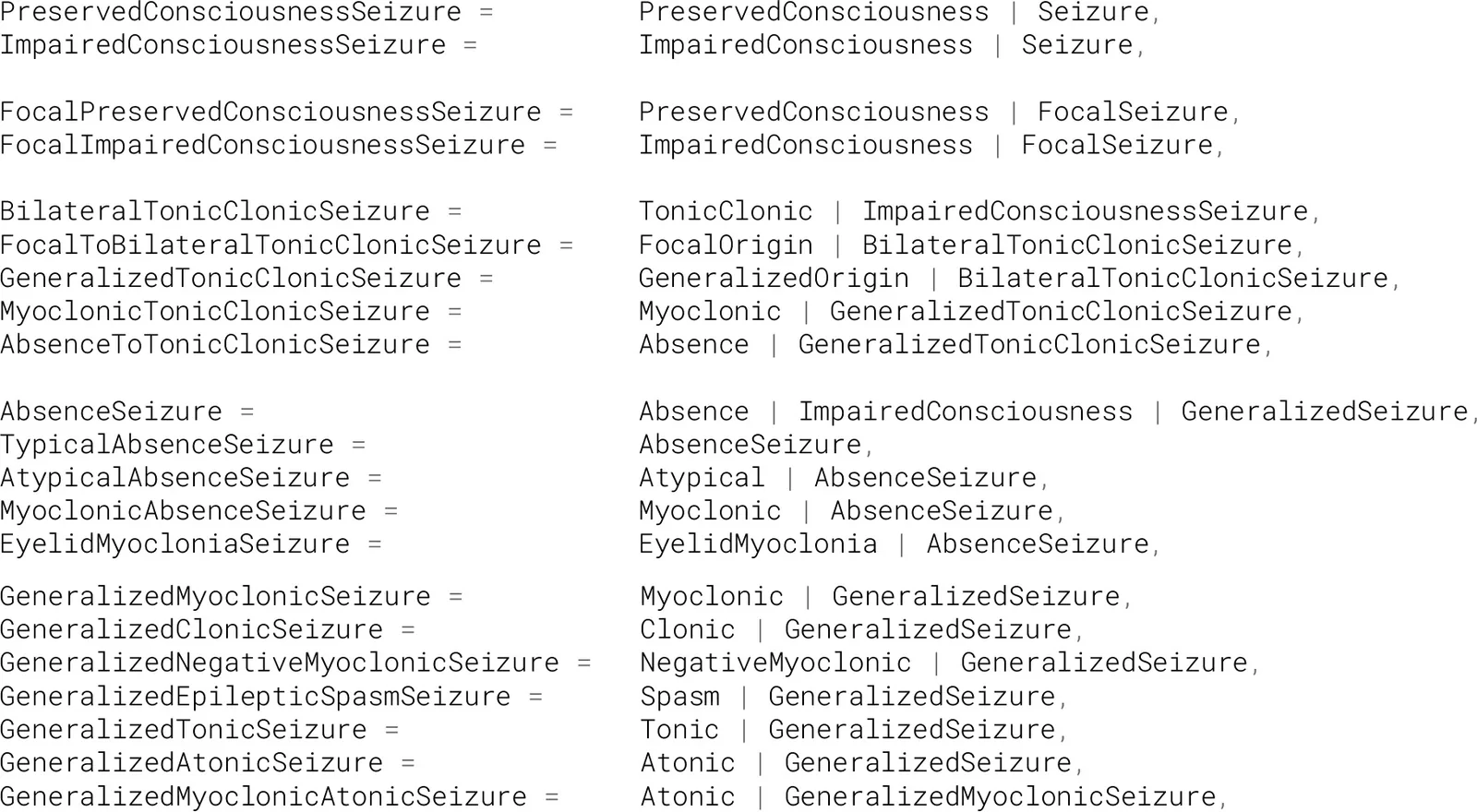


As ClassificationType now defines both flags and fully-specified “configurations”, it is not a well-constrained key for a map of seizure names (e.g., a list presented to a user). TypeScript helps us define a subset of these values that can then be used in other types. For example, both the canonical list of seizure types and their corresponding names can be combined into a map, providing improved type safety as any modifications to CanonicalClassificationType will require updating SeizureNames. Another advantage is that developers can index SeizureNames with a ClassificationType (without a cast), but the compiler will prevent statically indexing the map with a value not in the canonical list.

**Figure:**
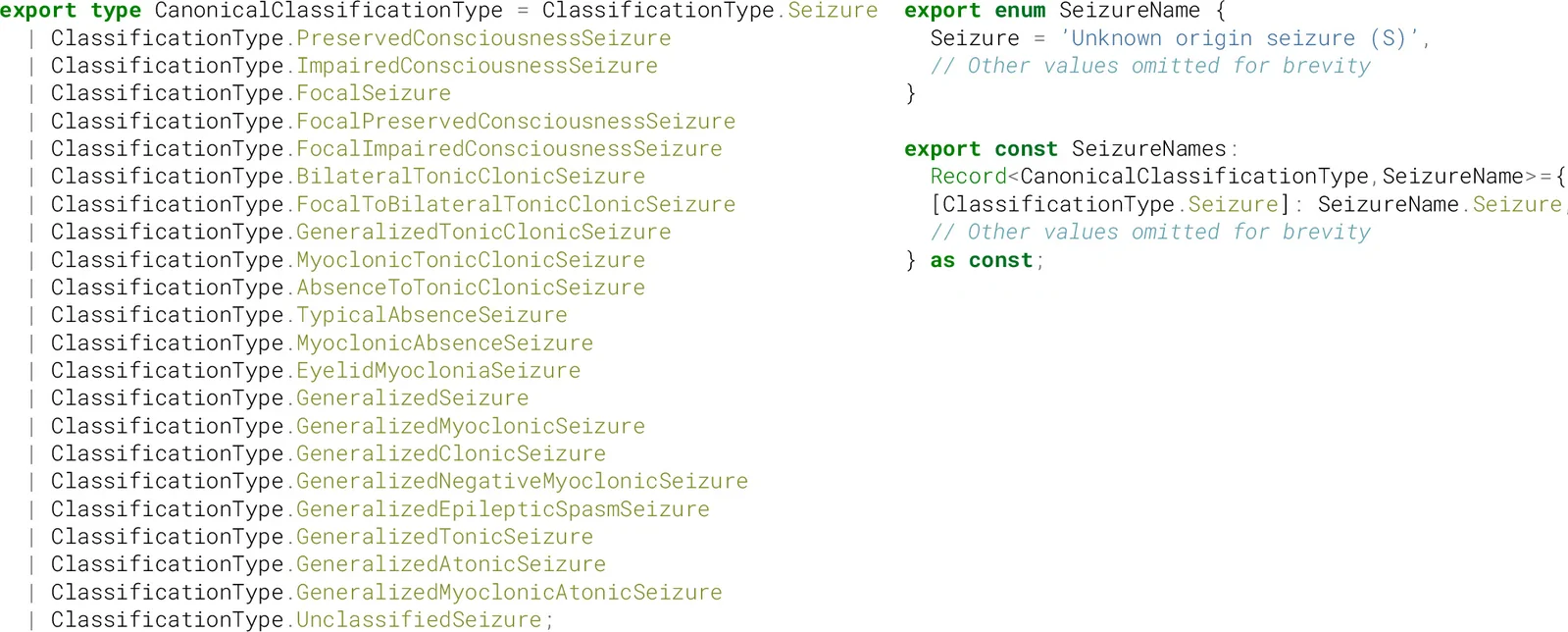


### 2.5 Expanding the basic classification with descriptors

Under the ILAE 2025 system, seizure classifications of the unknown and focal classes can be expanded through the use of semiological descriptors and somatotopic modifiers. The position paper states that “*[f]eatures directly impacting patient management [are] designated as classifiers, whereas other seizure characteristics [serve] as descriptors*”. As such, descriptors can be seen as not directly part of the seizure type, but are still valuable in documenting the seizure phenomenology. In addition to contributing to the expanded classification, they also are intended to capture the temporal sequence of the respective phenomena.

Numerically, we propose that descriptors extend the base classification model with unique numeric bit flags, inheriting existing values and reusing the base flags where appropriate to define expanded subtypes of seizure phenomena. This is especially appropriate given how the position paper states that “*motor phenomena defining generalized seizure types (myoclonic, tonic, atonic) may also be part of the semiology of a focal seizure*”. While this approach extends naturally with the small number of seizure type flags already defined, the one hundred predefined semiological groups and features do not fit into a bitfield of 32 bits when using one dedicated bit per feature. As both the position paper and practical guide state that “*if phenomena not listed above occur during the seizure, they are added in free text*”, it is implied that they may grow over time, or that users may need to define their own reusable descriptor definitions. Therefore, we propose defining only the somatotopic modifiers and top-level semiological group flags into the remaining initial 32 bits, and permitting the remaining semiological features to be encoded as values beyond the thirty-second bit. This results in the following definition for somatotopic and semiological feature group flags:

**Figure:**
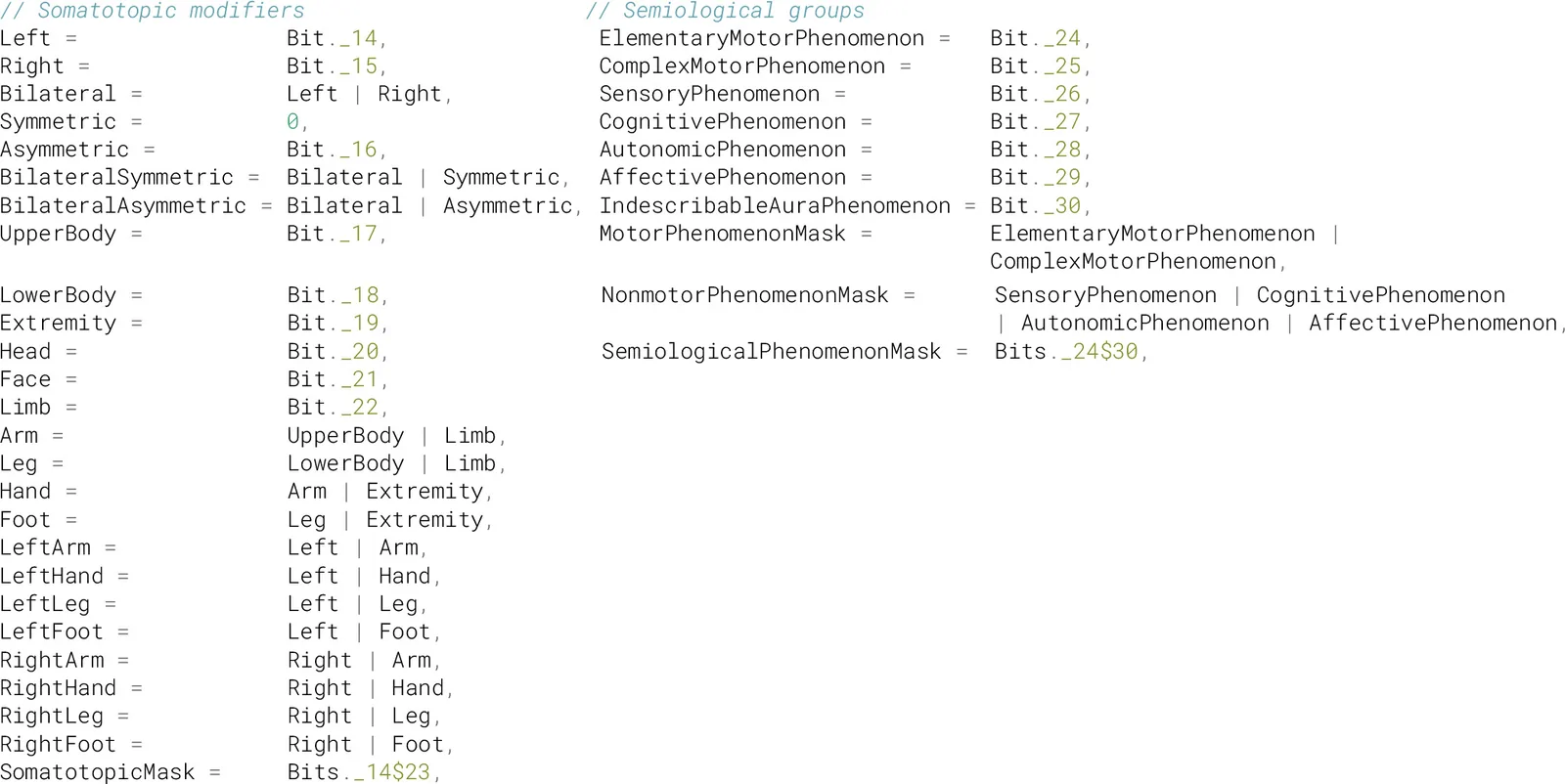


The body part and direction bits have been selected to be compact and associative, allowing for various degrees of freedom during selection within a small bit range. Similarly, the seven semiological groups are encoded in the final available bits, allowing for search cases by group.

The “concrete” descriptors are then created by ORing the group bit(s) with a phenomenon-specific bit. For book-keeping, an internal enumerated type Phenomenon is defined to ensure every phenomenon has an associated value. While there are one hundred predefined values in the position paper, reusing the lower bits from the phenomena listed in the basic ontology leads to an effective phenomenon count of 123. Thus, the combined (ORed) value for any combination of classification type and descriptors can fit into a 128-bit integer (while still leaving a few free bits for future extensions). As the descriptor bits are outside of the initial 32-bit range, big integers have been used to define these i TypeScript:

**Figure:**
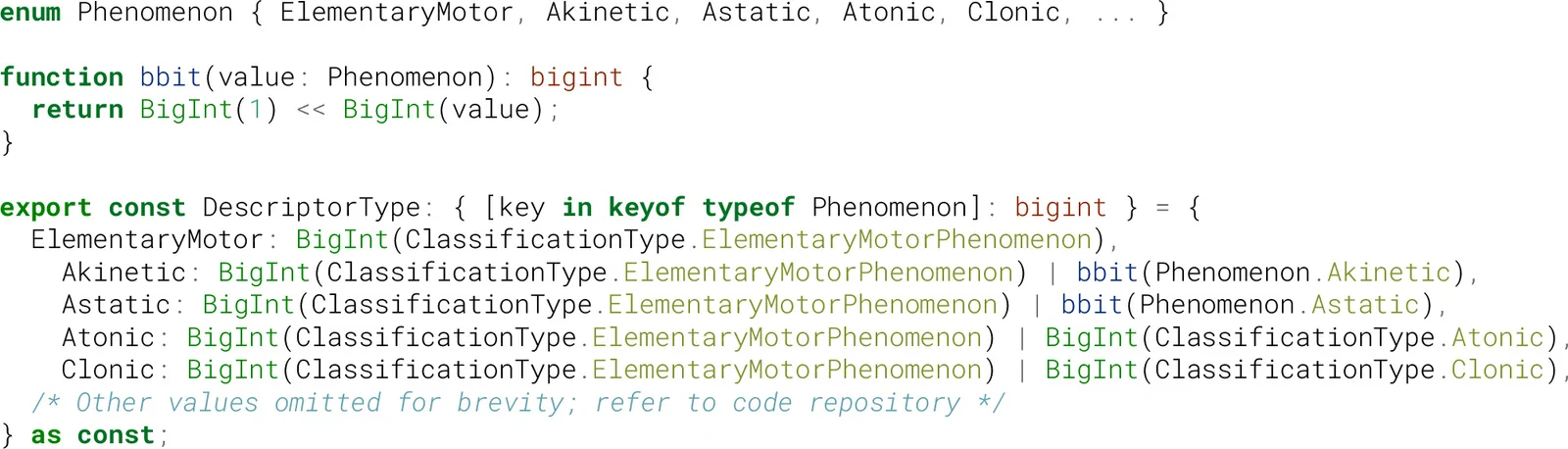


Returning to the earlier discussion about including Tonic and Clonic flags in the preset definition of a tonic-clonic seizure, consider the case where the seizure is initially of Unknown origin, but both tonic and clonic epochs are noted during the event. The clinician (or an algorithm) would attach these descriptors to the event, creating an Unknown seizure with Tonic and Clonic components. This is not equivalent to a TonicClonic seizure, as that bit is not set. Later, based on additional observable phenomena (such as head version) or other clinical evidence (e.g., EEG readings), the clinician (perhaps at the suggestion of an algorithm) might classify the seizure as a FocalToBilateralTonicClonicSeizure (thus setting the TonicClonic bit). The attached descriptors would remain, retaining valuable information about the precise moments when the semiological features occurred. The combined numerical value of the classification would inherit the canonical type (FocalToBilateralTonicClonicSeizure) and any bits from its attached descriptors (i.e., Versive | Head | Left | Tonic | Clonic).

As the practical guide states, “*[descriptors are] not necessary for generalized seizures, as the seizure type name already implies the key semiological features*”. However, no attempt has been made to enforce this constraint (i.e., dropping all descriptors when the seizure is generalized) in the current proposal, given that the clinical objectives of medical device software may vary considerably. For example, it may be useful for a system to offer the ability to attach descriptors to generalized seizures for the purposes of highlighting the temporal sequence of subevents. We also note in the Results section that this constraint may have a negative impact when offering the user the ability to switch between generalized and other seizure types during classification. In the example above, we would not necessarily want to “erase” the attached descriptors.

With this approach, all descriptor phenomena can be added to a list of all phenomena known to the application, whether ILAE-defined canonical phenomena or user/application-defined. When a phenomenon is nested within a group, its inheritance is preserved by ORing with the parent's phenomenon flag (just as done with the seizure types). For example, in the excerpt above, all values inherit ElementaryMotor, and this is based on the previously-defined ElementaryMotorPhenomenon. Similarly, the Atonic and Clonic descriptors also inherit their previously-defined values. For defining new bits, a helper function (bbit()) is used to assist with the left-shift big integer arithmetic, as illustrated by the Akinetic and Astatic examples. Following this pattern, all ten predefined postictal descriptors were added to the DescriptorType map, each inheriting a common Postictal bit. An implementation would then have the freedom to add an arbitrary number of additional phenomena simply by assigning them the next available serial number, while specifying direction and body part would be done by incorporating the bit flags for the respective somatotopic modifier(s).

As the position paper allows for free text to be used when describing the seizure semiology (particularly for phenomena not covered by the predefined values), the implementer might choose to either provide free text as a field in its descriptor class implementation, or require the creation of a reusable descriptor template with text that applies whenever an instance of the template is created. Furthermore, in order to model the temporal sequence of descriptors, the implementation should provide rank and/or timestamp fields for its descriptor object. While a complete description of the class structure is outside the scope of this study, it is important to highlight that these choices made in the underlying implementation will have a considerable effect on the available use cases.

#### 2.5.1 Big integer serialization notes

While the base classification is encoded within a 32-bit integer—a type with ubiquitous support across runtimes and frameworks—the use of big integers may require additional handling when exchanging between systems (such as writing to an API or storing in a database). Some implementers may have the option of using a binary representation or wire format (typically provided by a third-party library) that natively supports wide (at least 128-bit) integers (and can properly handle implementation details such as endianness). However, many application programming interfaces (APIs), such as those based on HL7 Fast Healthcare Interoperability Resources (FHIR) (HL7.org, 2023), will use a representation in a text-based format such as JavaScript Object Notation (JSON) or Extensible Markup Language (XML). JSON, for instance, does not natively support big integers; similarly, FHIR itself supports the decimal and integer64 types, but it has no support for wider integer types. One common convention is to “wrap” these values in a string, using the 0x-prefixed hexadecimal representation. While this approach may be inferior in both storage and computational efficiency when compared to pure binary representations, it has the likely benefit of being simpler to implement and more readily available in most modern programming contexts without the use of third-party libraries. To demonstrate, Table 2 considers this serialization strategy for various languages.

**Table 2:** Big integer data exchange via 0x-prefixed hexadecimal strings strategies for various runtimes.

Language	Native type	Serialization	Construction
JavaScript/TypeScript	BigInt	‘0x${v.toString(16)}’	BigInt(s)
Implementers can define BigInt.prototype.toJSON() in order to use JSON.stringify() directly, making serialization to JSON more convenient.			
Python	int	f'0x{v:x}' or hex(v)	int(s, 16)
Native Python integers have unlimited precision.			
Java	BigInteger	“0x” + v.toString(16)”	new BigInteger(s.replaceFirst (“^0x”, “”), 16)
Unlike the previous entries, Java requires stripping the prefix in order to deserialize.			
Rust	u128	format!(“0x{:x}”, v)	u128::from_str_radix(s.strip_prefix(“0x”).unwrap(),16)
Like Java, the hexadecimal prefix must be removed before conversion. Unlike the previous entries, uint128 is limited to 128 bits; arbitrary integer			
support is possible via a third-party crate.			
C/C++ (GCC)	__uint128_t	std::format(“0x{:x}”, v) (C++/libstdc++) / sprintf(x, “0x%lx%lx”, uint64_t(v >> 64), uint64_t(v)) (C)	__uint128_t(stoull(s.substr(0, s.size() - 16), nullptr, 16)) < < 64 | __uint128_t( stoull(s.substr(s.size() - 16), nullptr, 16))
As in Rust, the __uint128_t type is limited to 128 bits. It is also a non-standard extension, and compilers may lack support for directly declaring 128-bit values			
in code. As standard support is limited, constructing from multiple 64-bit components is required.			
SQL (PostgreSQL)	bit(128)	SELECT '0x' || encode(substr( bit_send(v), 5), 'hex')	select ('x' || LPAD(TRIM(LEADING '0x' FROM s), 32, '0'))::bit(128)
The numeric (decimal) type supports arbitrary precision, but does not support bitwise operators, so bit strings are more appropriate in practice. They			
can be correctly constructed from padded hexadecimal strings, but the reverse operation requires using a PostgreSQL-specific binary transmission function.			

The variables v and s represent a native value and a serialized value (of a number requiring at least 65 bits), respectively. Possible error handling has been omitted from the example expressions.

Python arguably provides the most elegant support, allowing for concise code and little room for error. JavaScript/TypeScript, Java (Oracle, 2026), and Rust (The Rust Team, 2026) come close in their developer ergonomics, as they all have native support for wider integer types and hexadecimal string encoding. Some C/C++ compilers, such as the GNU Compiler Collection (GCC), do include extensions for 128-bit integers (Free Software Foundation Inc., 2026), but such support may not be available on all platforms (nor with other compilers). As standard library functions do not support 128-bit values (with the notable exception of libstdc++'s std::format), additional numeric wrangling is likely required in practice. Standard SQL supports the bit type, and PostgreSQL has native support for bit string columns (The PostgreSQL Global Development Group, 2026), including integer-like bitwise operators for combination and search. It unfortunately requires a non-portable method for serializing the value to hexadecimal: as of PostgreSQL 18, it does not yet support casting from bit to to the binary blob type bytea, nor does it have a hexadecimal output format like it does for bytea. These examples show that, while big integer support is generally available in common runtimes, it may come with caveats that the implementer must keep in mind.

### 2.6 Extending the classification system

One of the main questions that we anticipate will arise, particularly in medical device software, is how to address the need to describe phenomena not present in the ILAE 2025 list of descriptors and event types. For example, and as we pointed out earlier, there is no provision for adding descriptors to generalized seizures, despite the fact that those of unknown (whether focal or generalized) class might initially have such descriptors attached. Therefore, there must be room not only to extend the classification system beyond the canonical definitions, but also to provide rules for migrating between types.

To address the first issue, we propose that an information system be permitted to use bit flags beyond those defined in the position paper to define extensions. We propose a “base class” for nonepileptic types with the name Unspecific:

**Figure:**
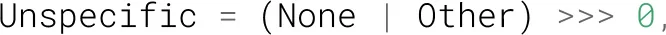


Effectively, this value is an alias for Other. However, unlike UnclassifiedSeizure, it does not inherit Epileptic. This is the starting point for implementing classifier and descriptor types that are not defined by ILAE 2025, but still operate within the same information framework in a compatible manner.

To address the second issue, we propose that an information system be permitted to store descriptors for any seizure class, as long as the canonical type can still be recovered and presented to the user. We consider this case with examples in the Results section.

## 3 Results

In order to verify the applicability of the specification, we may first consider the implications for implementation, using the code repository as the practical reference. With an established programming environment, it is then possible to explore the complete bit mapping of the prespecified seizure types defined in the ILAE 2025 position paper. As a natural extension, we have further mapped all the seizure types described in the ILAE 2017 commentary paper. From there, we also have examined the various bitwise search cases that might be used in practice. Finally, we apply the model to a selection of clinical vignettes to test its ability to capture the new standard. As a way to bring all these pieces together, an end-to-end web application example is demonstrated.

### 3.1 Coverage and quality metrics

The project has taken a number of steps to encourage code quality and correctness. It includes a 326-test conformance suite designed to test independent implementations against all known enumerated values, canonical seizure types, correct bitwise query results, and serialization. Each implementation is expected to read the input values (deserializing them from JSON), perform the requested task, and serialize the output matching the expected value. As of version 0.0.4 (Git commit 3e320f3c), conformance tests are available for Python and JavaScript, and these both run within GitLab continuous integration (CI) (GitLab Inc., 2026) with full coverage of the test suite. The Python conformance test is entirely independent of the TypeScript code, redefining all enumerated values and operations. As the JavaScript test is intended as a reference sample for writing future conformance tests, it reuses the project's exported type structures but still performs its own operations. While these conformance scripts are not intended to be complete reimplementations of the library, they are both evidence of cross-language interoperability and a potential starting point for developing a compatible implementation within a new environment.

Additionally, the project performs the following checks automatically in CI:

- Confirmation that the library and documentation builds
- Four Vitest (VoidZero Inc. and Vitest Contributors, 2026) suites with 665 total unit tests covering canonical seizure type collisions, inheritance structure, clinical examples, ILAE 2017 mapping, and validation functionality (including bitwise combination tests that address issues such as JavasScript's unsigned integer caveats)
- Code style enforcement via ESLint (OpenJS Foundation, 2026a).

These controls help validate the project's aims, while providing sustainable checks as the codebase evolves. As documented in the software repository, users can access the built-in tests after cloning the repository and running the following terminal commands:


yarn install
yarn test


A text-based test report is then printed to the console. In merge requests, this information is also available within the GitLab user interface.

### 3.2 Validating encodings

Depending on the situation where the proposed encoding is used, not all bit combinations may be valid. Concretely, the definitions describe a set of masks that, under certain conditions, must be constrained to either a specific bit or bit pattern. Table 3 summarizes the number of valid bits within each mask (following the masks' intended semantics), allowing for numeric validation based on the bit population count of that mask (and the target use case).

**Table 3:** Value constraints for each bitmask: the number of allowable bits within each mask can be validated per use case.

Bitmask	Bit width	Canonical classification^a^	Classification	Canonical descriptor^a^	Descriptor	Combination
Seizure	1	== 1	<= 1	== 0	<= 0	<= 1
Other	1	<= 1	<= 1	<= 1	<= 1	<= 1
OriginMask	2	<= 1	<= 1	== 0	== 0	<= 1
ConsciousnessMask	2	<= 1	<= 1	<= 1	<= 1	<= 2
TraitMask ^b^	9	<= 2	<= 9	<= 2	<= 9	<= 9
SomatotopicMask ^b^	10	== 0	<= 10	<= 1	<= 10	<= 10
SemiologicalPhenomenonMask ^b^	7	== 0	<= 7	<= 1	<= 7	<= 7
Postictal ^c^	1	== 0	== 0	<= 1	<= 1	<= 1

^a^The canonical classifications and descriptors are validated by checking if they exist in the CanonicalClassificationType and DescriptorType sets defined in §2.4.4 and §2.5. Therefore, the values in these columns are a consequence of those definitions rather than limits imposed by the semantics of the corresponding mask.

^b^While not clinically likely, there is no specific maximum for the number of traits, somatotopic groups, or semiological phenomena applied to custom classifications/descriptors. If applicable, detecting “oversaturated” masks or unlikely combinations should performed by application-level heuristics.

^c^The Postictal bit is only defined on DescriptorType, and therefore can only appear in descriptors and combinations.

To aid implementers in adhering to these constraints, a validate() function has been included in the project API. It takes a single numeric value (a Number or BigInt), optionally a validation type (defaulting to CanonicalType), and returns a structure with the following shape:

**Figure:**
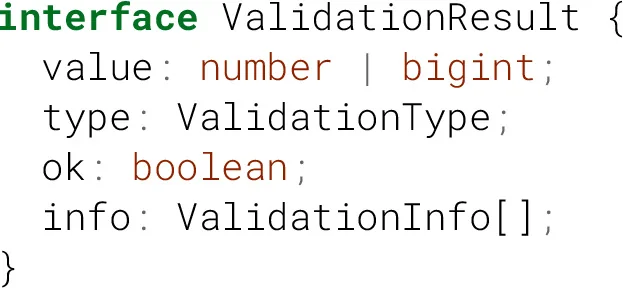


The function can be used in the following scenarios, as determined by the ValidationType passed to the function:

- ValidationType.CanonicalClassification: this checks that the value is one of the 26 canonical seizure types defined in the position paper.
- ValidationType.CanonicalDescriptor: this checks that the value is one of 131 descriptors and sematotopic modifiers defined in the position paper.
- ValidationType.Classification: this checks that no known conflicts within masks have been identified, when considering the value to be a valid classification. This can be considered a check that the type represents a potentially valid “partial” or “custom” classification (meaning that while not canonical, it does not break any clinical rules). For example, specifying both Generalized and Focal or both ImpairedConsciousness and PreservedConsciousness within the same value would lead to an invalid result.
- ValidationType.Descriptor: this checks that no known conflicts within masks have been identified when considering the value to be a single descriptor, such as using multiple semiological phenomenon groups. This is intended to test for potential logical flaws when combining descriptor bits.
- ValidType.Combination: this considers checks that only apply when the classification and descriptor(s) are combined in a single value, running only the checks from above that apply within this scenario. For example, the origin check applies because individual seizures are not expected to be described with both foci. However, the consciousness and semiological group checks are not run, as complex cases may describe seizures that contain traits from several mutually-exclusive groups.

The developer is expected to check the result's ok value to determine if the check passed. The info member is an array of ValidationInfo values—predefined enumerated strings—that can be used to handle the specific type of validation issue(s) found. These strings may be displayed in a user interface, for example, if the end-user is expected to make a correction.

### 3.3 Implementation checklist

Implementers can already use the reference code repository to integrate the proposed model, noting that the offering (as of version 0.0.4) remains limited in scope:

- Define the identifiers (names) and associated values of all seizure and descriptor traits defined in the 2025 position paper.
- Provide utility methods and example code for using these values within a TypeScript/JavaScript application.
- Use a unit testing framework to ensure proper behavior of the proposed encoding across multiple use cases and clinical criteria.

Accordingly, the project does not currently offer any of the following (although they may be added in the future):

- Implementation for any other programming languages or frameworks other than TypeScript/JavaScript
- Pre-built interfaces/classes for managing seizure and/or descriptor objects
- Mapping to/from other interoperability ecosystems
- Built-in support for “plugins” or other extension systems for adding new event types or descriptors (e.g., for non-epileptic seizures).

It is up to the implementer to make the downstream design choices (such as building interfaces around the proposed encoding) that are relevant to their application. For a TypeScript (or JavaScript) project, the implementer will be required to:

1. Add the package as a dependency using the package manager of choice (npm, pnpm, yarn, etc.). The package has no runtime dependencies.
2. In the code file where seizure types are needed, import the relevant types (ClassificationType, DescriptorType, etc.) and possibly the validate() function. Code from the examples directory may guide usage.
3. Depending on the required actions of the application, apply bitwise arithmetic to combine or mask these values.
4. Use the validate() function to check for invalid or potentially-conflicting combinations as needed.
5. Store the validated numeric values in a manner specific to the application (e.g., within an SQL database).

The last component of the checklist is highly application-specific. Some types of applications may require advanced logic, especially when considering the handling of descriptors. Descriptors, from the position paper's perspective, are indeed *optional*, as they basic seizure model can be used where sufficient for clinical use. Attempting to implement support for ordered, or even timestamped and duration-annotated descriptors, with free text support, will increase the implementation complexity. Implementers must consider at least the following:

- Should parts (e.g., certain bitmasks such as origin and consciousness where zero is meaningful) be considered optional instead of inheriting the default value? In other words, is UnclassifiedSeizure a reasonable alternative, or do these values need to be tracked explicitly?
- Are individual descriptors supported? Are they stored as their own entities (e.g., as individual rows within the database)?
- Do descriptors require an order within the seizure? Do they require a rank or timestamp?
- Is free text supported? Will the free text be harmonized by the application to ensure semantic identity (e.g., by a large language model)?

Considering opposite ends of these requirement lists, two possible data models are described in Figure 1: a simple model containing only a combined numeric value, and a complex model supporting shared classifications and separable, ordered descriptors. Both models support a combined type value and optional free text. In the simple model, an event only needs to store a 128-bit integer type, but all separation of the classification and descriptors would be the responsibility of the application to parse (e.g., by using bitwise population count to identify the most specific canonical seizure type described by the value) and visualize (e.g., by using checkboxes or a multi-select field). The complex data model offers a more complete solution, as it provides for tracking of set bitmask values, explicit selection of the base classification, descriptor ordering, and per-descriptor free text. Notably, it stores classification and descriptors separately, with the type property derived from the two (presumably, by ORing all values together). To facilitate tracking of whether origin and consciousness have been explicitly set within the value, boolean values have been defined (these could alternatively be stored together as a single unsigned integer); conceivably, a custom getter could return true for these if the value's respective bitmasks have been populated, or otherwise return the internal state explicitly tracked by the setter. The optional rank and range (defined here as the modern replacement for JavaScript's Date object) would be used for specifying evolution order, start, and duration characteristics. While a concrete reference implementation of the seizure/descriptor programming model is not in scope of the project at this time, the implications of these two example data models can still be explored within the following sections.

**Figure 1:**
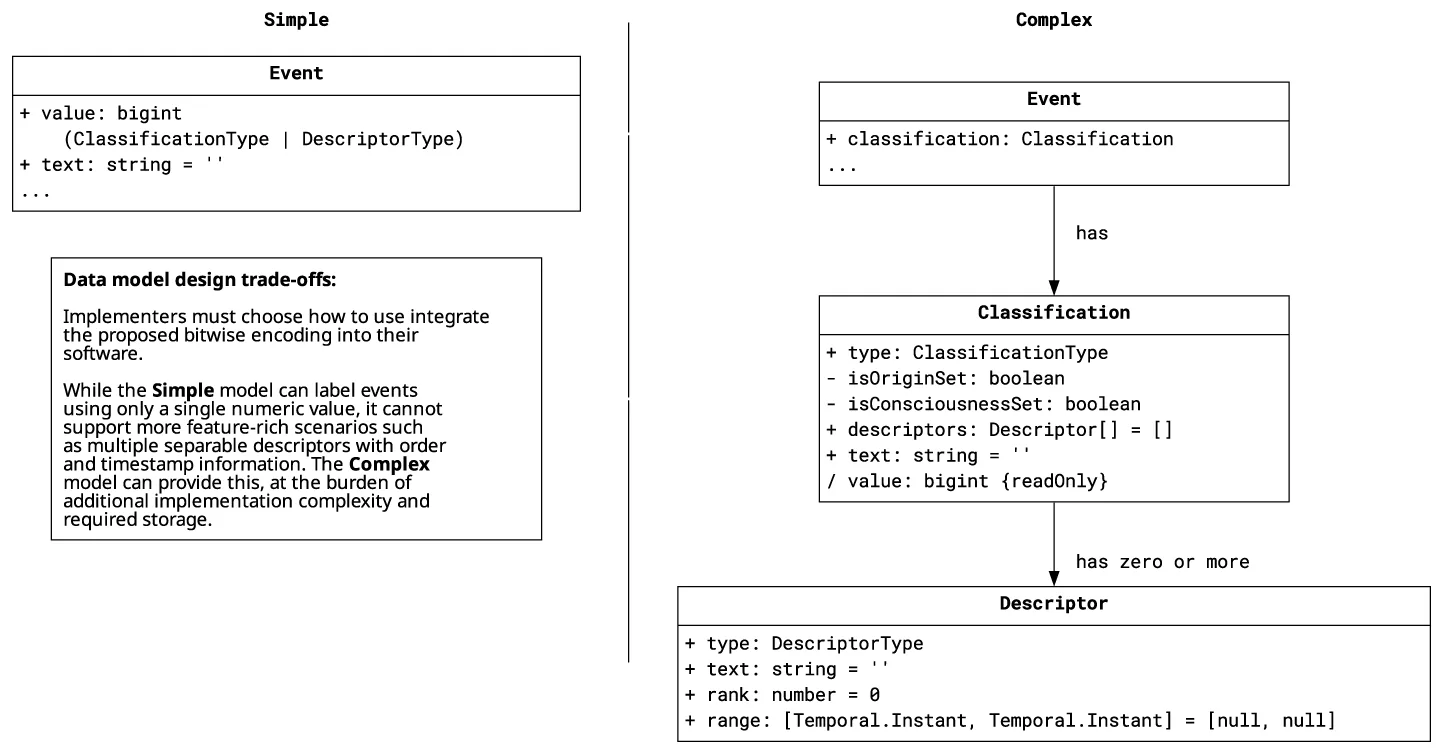
Potential data models utilizing the proposed bitwise encoding.

### 3.4 Visualizing the hierarchy

Given the configurations described above, we can visualize the base system ontology as a hierarchy, or inheritance tree (Figure 2). If we frame the hierarchy in terms of object-oriented polymorphism, we can think of the type traits as “interfaces” or “abstract” classes: they are inherited, but are not instantiated on their own. As the position paper states that “*classification of an individual seizure can halt at any level on the hierarchical tree*”, classes such as GeneralizedSeizure are available as a concrete type even when more specific subtypes are available. Some notable effects arise from the numeric choices made above:

- The most fundamental case is Epileptic: it is not a concrete (fully-defined) type, but it is inherited (either directly or indirectly) by all types in the hierarchy. While numerically speaking they are the same value in the current proposal, the TypeScript definitions impart the semantics that the type trait is inherited.
- All seizure types inherit (either directly or indirectly) the Seizure type. Without any further classifiers, this corresponds to a seizure that is unknown whether focal or generalized. Using this model, the UnclassifiedSeizure then becomes a “subclass” of this type. While this may appear to be clinically surprising (as the Unknown classifier is arguably more specific than Unclassified), it is numerically intuitive: as UnclassifiedSeizure does not have a known origin, it naturally inherits the Seizure type.
- The AbsenceSeizure type has a similar pattern as Epileptic: while the position paper lists it as a grouping type for absence seizures (and ostensibly a concrete type), the position paper suggests that a more specific subtype will be found in practice. Therefore, it is a numerical choice (in order to save a bit) to treat TypicalAbsenceSeizure as the same value. As an absence that is not further specified would be assumed to be a typical absence seizure, the software interface would not allow selection of the more abstract AbsenceSeizure type. A potential workaround would be to use the ImpairedConsciousnessSeizure and attach a descriptor with the Absence bit, but this would be an extension to the canonical seizure list as absence is not a predefined semiological descriptor (while the proposed specification allows this, because the descriptor list extends the classification list). A GeneralizedSeizure with an Absence descriptor might be considered an even better substitute, at the cost of stretching the boundaries of the position paper even further. Assuming the application allows it, there is always the option of adding free text when a more suitable predefined descriptor is unavailable.
- There is no numerical distinction between EyelidMyocloniaSeizure with or without absence (EMA), as the position paper describes these as the same type. This is consistent with the finding that such events are often considered to be subclinical when absence is not present (Smith et al., 2023), and the 2025 position paper expressly avoids classifying subclinical events. So while there is a numerical solution to encoding an “eyelid myoclonia seizure without absence” type (i.e., by clearing the Absence bit), it is not a canonically-defined type.

**Figure 2:**
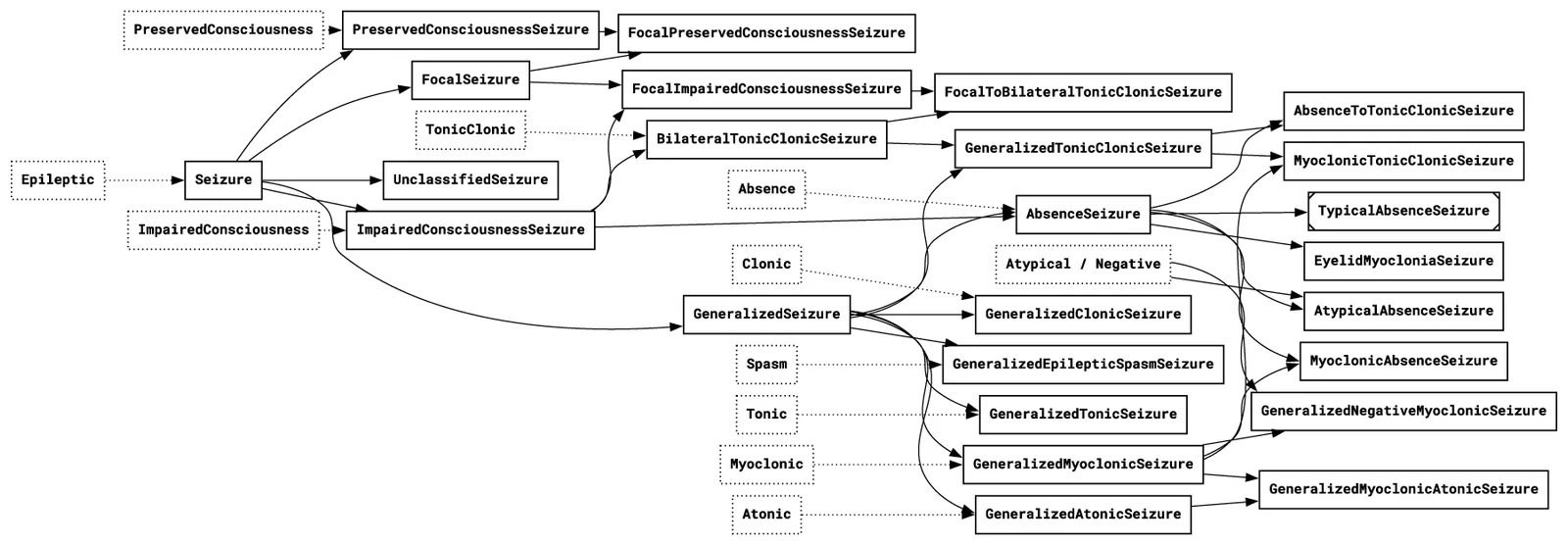
Inheritance diagram for canonical ILAE 2025 seizure types. Concrete seizure types are represented as solid boxes; abstract type traits are represented as dotted boxes. TypicalAbsenceSeizure is marked with diagonal corners to highlight that it is numerically equivalent to its inherited AbsenceSeizure “parent class” within the proposed model.

The complete bitfield patterns, along with the extensions mentioned, are shown in Table 4.

**Table 4:** The bit mapping of seizure types to traits.

Type Trait (bit)	Epileptic (0)	Focal origin (1)	Generalized origin (2)	Preserved consciousness (3)	Impaired consciousness (4)	Tonic-clonic (5)	Atypical / negative (6)	Absence (7)	Myoclonic (8)	Eyelid myoclonia (9)	Clonic (10)	Spasm (11)	Tonic (12)	Atonic (13)	(Somatotopic/Semiology bits)	Other (31)	Hexadecimal
No information																	0x00000000
Unknown origin seizure (U)																	0x00000001
Preserved consciousness (PC)																	0x00000009
Impaired consciousness (IC)																	0x00000011
Bilateral tonic-clonic (BTC)																	0x00000031
Focal seizure (F)																	0x00000003
Focal preserved (FPC)																	0x0000000b
Focal impaired (FIC)																	0x00000013
Focal to bilateral tonic-clonic (FBTC)																	0x00000033
Generalized seizure (G)																	0x00000005
Generalized tonic-clonic (GTC)																	0x00000035
Myoclonic tonic-clonic (GMTC)																	0x00000135
Absence to tonic-clonic (GATC)																	0x000000b5
Typical absence (TA)																	0x00000095
Atypical absence (AA)																	0x000000d5
Myoclonic absence (MA)																	0x00000195
Eyelid myoclonia (EMA)																	0x00000295
Generalized myoclonic (GM)																	0x00000105
Generalized clonic (GC)																	0x00000405
Generalized negative myoclonic (GNM)																	0x00000145
Generalized epileptic spasms (GES)																	0x00000805
Generalized tonic (GT)																	0x00001005
Generalized atonic (GA)																	0x00002005
Generalized myoclonic-atonic (GMA)																	0x00002105
Unclassified seizure																	0x80000001
Unspecific (unconfirmed epileptic)																	0x80000000

Epileptic extensions can be made by toggling bits on the predefined values, but may not always result in a clinically-meaningful result. Nonepileptic extensions can be made by inheriting “Unspecific” without including the “Epileptic” trait. Dark gray cells indicate that the bit is set (1). Light gray cells indicate that the bit is unset (0).

### 3.5 Migrating between types in practice

Clinical classification (whether entirely human-provided or assisted by artificial intelligence) is likely to take an additive approach: as more evidence becomes available, the classification is refined. Therefore, a classified event label may pass through various stages (numerical identities) over the course of the clinical workflow. From the view of the information system, this can be viewed as a migration: a systematic set of operations that occurs “under the hood” when a classification action is performed.

Consider the example where an application interface allows the user to transition a label from “Unknown (whether focal or generalized) seizure with myoclonic activity” to a “Generalized myoclonic seizure”. A possible implementation might proceed as follows:

1. The system's database has two objects: a Classification of type ClassificationType.Seizure with an attached Descriptor object of type DescriptorType.Myoclonic.
2. When the user changes the type to “Generalized Myoclonic” in the user interface, the system effectively changes the classification's type to ClassificationType.GeneralizedMyoclonic.
3. As the Descriptor object is no longer required (because the seizure type is generalized), it is deleted.

This implementation takes a “strict” approach to adherence with the standard by dropping the ostensibly superfluous descriptor. If the descriptor truly holds no information, such as precise timestamp information, no information is lost in this transition. If those constraints hold for the application, the same result might be achieved with an even simpler approach:

1. The Classification object is implemented with a single type value, set to ClassificationType.Seizure | ClassificationType.Myoclonic. This is interpreted, by virtue of the UnknownOrigin, by the application interface as a seizure of unknown origin with a myoclonic descriptor (rather than as a singular canonical seizure type).
2. The classification's ClassificationType.Generalized flag is toggled on, resulting in a final type of ClassificationType.GeneralizedMyoclonicSeizure. The user interface no longer displays any descriptors, as the myoclonic aspect is part of the classification type.

Depending on the requirements of the software, either implementation above may be appropriate. While the second implementation is simpler (it requires fewer operations and does not model descriptors as separate objects), it may require additional parsing rules to correctly display the canonical seizure type. However, the second implementation has a distinct disadvantage: multiple Myoclonic descriptors cannot be modeled. As the first implementation stores the descriptor as a separate object, it might also be able to attach timestamp information, allowing the application to model an entire series of myoclonic subevents within a myoclonic seizure cluster. However, this also implies that such descriptors should not be discarded when the classification is changed to a generalized seizure type: doing so would erase valuable clinical information. Furthermore, it is often important that medical software retain an edit history or audit trail visible to the user; in this example, it may be clinically relevant to know at what point the classification was resolved to generalized epilepsy. Therefore, it is important for medical software to distinguish between what is required by the standard and what additional capabilities (such as retaining descriptors for generalized seizures) is required by its users.

### 3.6 Mapping from ILAE 2017 to ILAE 2025

In a brief commentary on the new classification system (Beniczky et al., 2025c), the authors state that “*[t]he fundamental structure of the classification remains unchanged, and databases using the 2017 version can be easily converted to the updated classification*”. Following similar logic as the internal migration between types, we may test this assertion by defining rules to map between 2017 and 2025 editions of the guidelines, using the list of 63 coded seizure types proposed by the SCORE consortium (Beniczky et al., 2017) as the original canonical list:

1. If the seizure has been marked as focal, generalized, or unknown *onset*, start with the corresponding FocalSeizure, GeneralizedSeizure, or Seizure type. The “Unclassified seizure (III.C.01)” coded type could map to either Seizure (of unknown origin) or UnclassifiedSeizure; this is discussed further below. If there are no consciousness or behavioral traits, the seizure is already fully specified.
2. If the seizure has been marked as preserved or impaired awareness, mark PreservedConsciousness or ImpairedConsciousness, respectively. In the ILAE 2017 guidelines, only seizures with a focal onset should have these specifiers. If there are no behavioral traits, these seizures are now fully specified.
3. If the seizure is tonic-clonic, mark the TonicClonic bit. If the seizure is myoclonic-tonic-clonic (II.A.04), also mark the Myoclonic bit (note that absence-to-tonic-clonic was not a recognized subtype in ILAE 2017). If there are no other descriptive features, such seizures are now fully specified.
4. For seizures of the Generalized origin, mark the corresponding bit if the seizure has a type trait listed in Table 5. These seizures are now fully specified.
5. For focal and unknown onset seizures with a coded type, attach the corresponding descriptor(s). These seizures are now fully specified.

**Table 5:** Mapping from ILAE 2017 defined types to 2025 defined types (with descriptors).

2017 Code	2017 Name	2025 Code	Enumerated descriptor(s)	Hexadecimal	Notes
I.A.01	Focal aware motor-onset seizure	FPC	MotorPhenomenonMask	0x0000000300000b	1, 2
I.A.02	Focal aware myoclonic seizure	FPC	MyoclonicPhenomenon	0x0000000100010b	1
I.A.03	Focal aware clonic seizure	FPC	ClonicPhenomenon	0x0000000100040b	1
I.A.04	Focal aware tonic seizure	FPC	TonicPhenomenon	0x0000000100100b	1
I.A.05	Focal aware atonic seizure	FPC	AtonicPhenomenon	0x0000000100200b	1
I.A.06	Focal aware automatism seizure	FPC	AutomatismPhenomenon	0x0010000200000b	1
I.A.07	Focal aware hyperkinetic seizure	FPC	HyperkineticPhenomenon	0x2000000200000b	1
I.A.08	Focal aware nonmotor onset seizure	FPC	NonmotorPhenomenonMask	0x0000007c00000b	1, 2
I.A.09	Focal aware behavior arrest seizure	FPC	MotorAndBehavioralArrest	0x0002000100000b	1
I.A.10	Focal aware sensory seizure	FPC	SensoryPhenomenon	0x0000000400000b	1
I.A.11	Focal aware emotional seizure	FPC	AffectivePhenomenon	0x0000002000000b	1
I.A.12	Focal aware cognitive seizure	FPC	CognitivePhenomenon	0x0000000800000b	1
I.A.13	Focal aware autonomic seizure	FPC	AutonomicPhenomenon	0x0000001000000b	1
I.A.14	Focal aware seizure	FPC		0x0000000000000b	
I.B.01	Focal impaired awareness motor onset seizure	FIC	MotorPhenomenonMask	0x00000003000013	1, 2
I.B.02	Focal impaired awareness myoclonic seizure	FIC	MyoclonicPhenomenon	0x00000001000113	1
I.B.03	Focal impaired awareness clonic seizure	FIC	ClonicPhenomenon	0x00000001000413	1
I.B.04	Focal impaired awareness tonic seizure	FIC	TonicPhenomenon	0x00000001001013	1
I.B.05	Focal impaired awareness atonic seizure	FIC	AtonicPhenomenon	0x00000001002013	1
I.B.06	Focal impaired awareness automatism seizure	FIC	AutomatismPhenomenon	0x00100002000013	1
I.B.07	Focal impaired awareness hyperkinetic seizure	FIC	HyperkineticPhenomenon	0x20000002000013	1
I.B.08	Focal impaired awareness nonmotor onset seizure	FIC	NonmotorPhenomenonMask	0x0000007c000013	1, 2
I.B.09	Focal impaired awareness behavior arrest seizure	FIC	MotorAndBehavioralArrest	0x00020001000013	1
I.B.10	Focal impaired awareness sensory seizure	FIC	SensoryPhenomenon	0x00000004000013	1
I.B.11	Focal impaired awareness emotional seizure	FIC	AffectivePhenomenon	0x00000020000013	1
I.B.12	Focal impaired awareness cognitive seizure	FIC	CognitivePhenomenon	0x00000008000013	1
I.B.13	Focal impaired awareness autonomic seizure	FIC	AutonomicPhenomenon	0x00000010000013	1
I.B.14	Focal impaired awareness seizure	FIC		0x00000000000013	
I.C.01	Focal motor seizure	F	MotorPhenomenonMask	0x00000003000003	1, 2
I.C.02	Focal myoclonic seizure	F	MyoclonicPhenomenon	0x00000001000103	1
I.C.03	Focal clonic seizure	F	ClonicPhenomenon	0x00000001000403	1
I.C.04	Focal epileptic spasm	F	SpasmPhenomenon	0x00000001000803	1
I.C.05	Focal tonic seizure	F	TonicPhenomenon	0x00000001001003	1
I.C.06	Focal atonic seizure	F	AtonicPhenomenon	0x00000001002003	1
I.C.07	Focal automatisms seizure	F	AutomatismPhenomenon	0x00100002000003	1
I.C.08	Focal hyperkinetic seizure	F	HyperkineticPhenomenon	0x20000002000003	1
I.C.09	Focal nonmotor seizure	F	NonmotorPhenomenonMask	0x0000007c000003	1, 2
I.C.10	Focal behavior arrest seizure	F	MotorAndBehavioralArrest	0x00020001000003	1
I.C.11	Focal sensory seizure	F	SensoryPhenomenon	0x00000004000003	1
I.C.12	Focal emotional seizure	F	AffectivePhenomenon	0x00000020000003	1
I.C.13	Focal cognitive seizure	F	CognitivePhenomenon	0x00000008000003	1
I.C.14	Focal autonomic seizure	F	AutonomicPhenomenon	0x00000010000003	1
I.C.15	Focal seizure	F		0x00000000000003	
I.D.01	Focal to bilateral tonic-clonic seizure	FBTC		0x00000000000033	
II.A.01	Generalized motor seizure	G		0x00000000000005	
II.A.02	Generalized myoclonic seizure	GM		0x00000000000105	
II.A.03^†^	Myoclonic-atonic seizure	GMA		0x00000000002105	
II.A.04	Myoclonic-tonic-clonic seizure	GMTC		0x00000000000135	
II.A.05	Generalized clonic seizure	GC		0x00000000000405	
II.A.06	Generalized epileptic spasm	GES		0x00000000000805	
II.A.07	Generalized tonic seizure	GT		0x00000000001005	
II.A.08	Generalized atonic seizure	GA		0x00000000002005	
II.A.09	Generalized tonic-clonic seizure	GTC		0x00000000000035	
II.B.01	Typical absence seizure	TA		0x00000000000095	
II.B.02	Atypical absence seizure	AA		0x000000000000d5	
II.B.03	Myoclonic absence seizure	MA		0x00000000000195	
II.B.04	Eyelid myoclonia with absence / without absence	EMA		0x00000000000295	
III.A.01	Tonic-clonic seizure of unknown onset	BTC		0x00000000000031	
III.A.02	Epileptic spasm with unknown onset	U	SpasmPhenomenon	0x00000001000801	1
III.A.03	Motor seizure with unknown onset	U	MotorPhenomenonMask	0x00000003000001	1, 2
III.B.01	Behavioral arrest with unknown onset	U	MotorAndBehavioralArrest	0x00020001000001	1
III.B.02	Nonmotor seizure with unknown onset	U	NonmotorPhenomenonMask	0x0000007c000001	1, 2
III.C.01	Unclassified seizure	U		0x00000000000001	3

The combined binary values are listed in hexadecimal.

Note 1: Several seizure types (such as focal seizure types with a behavioral trait) have been simplified in ILAE 2025. These ILAE 2017 types can only be represented by combining a canonical classification with the relevant descriptor.

Note 2: ILAE 2025 no longer defines a generic nonmotor/motor components, but rather defines separate elementary and complex motor phenomena. As a consequence, these types must be mapped to a more specific value within the Nonmotor/Motor phenomenon masks in order to remain clinically correct. This may require access information external such as free text notes.

Note 3: The 2017 “Unclassified seizure” type could map to either the 2025 “Unknown” or “Unclassified” classes depending on available additional information.

^†^This value was printed in the original article as II.A.04, presumably due to a typographical error.

Following these rules, Table 5 maps all coded ILAE 2017 types into their corresponding ILAE 2025 types.

Reassuringly, all ILAE 2017 types (when considered with their descriptor flags) map to reasonable and unique values in ILAE 2025, and all hexadecimal mappings fit within the first 32 bits. However, as noted in the table, two important caveats must be observed:

- For all types in the table that include descriptors, the 2025 base (canonical) classification is not alone sufficient to map back to ILAE 2017—the entire combination must be used. This is because the simplified 2025 model removed many seizure types, delegating these “subtypes” to descriptors instead.
- The 2017 “motor-onset” and “nonmotor” types can only be completely specified through the use of more specific semiological descriptors from the observable and non-observable features. Implementers should be aware of this limitation and implement further descriptor matching based on other information (e.g., free text) that is already in their information system. By selecting at least one value within the target mask, the value remains unambiguously reversible to ILAE 2017. However, using an entire mask, while functionally valid, would likely lead to clinically incorrect documentation. Therefore, it is important that, prior to migration of an existing dataset, all ambiguous motor/nonmotor cases are processed using additional input or flagged for manual review.

With these two constraints understood, the mapping can be used losslessly.

The “III.C.01 Unclassified seizure” also has a somewhat ambiguous mapping, as the 2017 instruction manual advises:

The term unclassified comprises both seizures with patterns that do not fit into the other categories or seizures presenting insufficient information to allow categorization (p. 533).

A seizure might be unclassified due to inadequate information or inability to place the seizure in other categories (p. 536).

Effectively, this type can refer to cases where there is either inadequate medical information to corroborate the finding (mapping to the 2025 edition's Unclassified) or simply that no existing category is suitable despite having corroborating clinical evidence (mapping better to 2025's Unknown class). Therefore, implementers and clinical workers should consider all available information when mapping events of this code. Moreover, as the 2025 Unclassified type is intended as a temporary state, that aspect might be used as an indicator when mapping: for an ongoing study, mapping to UnclassifiedSeizure might be appropriate as it would encourage the clinical team to seek a more specific classification; for a historical record, Seizure (of unknown origin) may be a better fit because the record has ostensibly been reviewed and already enriched with the available clinical evidence.

### 3.7 Bitwise search cases

To evaluate the claim that the proposed structure offers a compact, efficient, and reliable documentation mechanism, a number of cases have been examined. Each case consists of a goal (e.g., find all seizures of a given type) defined by a search scope (a classification, descriptors, or the combined value) and a bitwise operand with comparator. The search scope can be the classification type alone, the descriptors individually (meaning a match on any of the descriptors), or the combined value. The operand is shown in Table 6 as the variable s; the result is all the values (e.g., database rows) for which the operation evaluates to true. It is worth noting that because the comparison operators have higher precedence than the bitwise operators, the bitwise operation must be placed between parentheses when used in a comparison.

**Table 6:** Various bitwise search cases for discovering ILAE 2025 labeled seizures from a database.

Search case	Scope (s)	Operation	Expected result
Seizures	Classification	(s & Epileptic) === Epileptic	Labels with the Epileptic bit
Focal seizures	Classification	(s & OriginMask) === FocalOrigin	Labels with the FocalOrigin bit
Generalized seizures	Classification	(s & OriginMask) === GeneralizedOrigin	Labels with the GeneralizedOrigin bit
Seizures of unknown (whether focal or generalized) origin	Classification	(s & (OriginMask | Other)) === UnknownOrigin	Labels of UnknownOrigin without the Other bit
Unclassified seizures	Classification	(s & UnclassifiedSeizure) === UnclassifiedSeizure	Labels containing all UnclassifiedSeizure bits, i.e. Seizure with the Other bit
Non-epileptic events	Classification	!(s & Epileptic)	Labels without the Epileptic bit
Events with impaired consciousness	Combination	(s & ImpairedConsciousness) === ImpairedConsciousness	Labels with the ImpairedConsciousness bit or descriptor
Tonic-clonic events (including subtypes)	Classification	(s & TonicClonic) === TonicClonic	Labels with the TonicClonic bit
Events with a tonic-clonic descriptor	Descriptors	(s & TonicClonic) === TonicClonic	Labels containing a TonicClonic descriptor
Events with a tonic-clonic bit	Combination	(s & TonicClonic) === TonicClonic	Labels containing a TonicClonic bit or descriptor
Only tonic-clonic events (without subtypes)	Classification	(s & TraitMask) === TonicClonic	Labels with only the TonicClonic trait bits
Generalized tonic-clonic seizures	Classification	(s & GeneralizedTonicClonicSeizure) === GeneralizedTonicClonicSeizure	Labels containing all GeneralizedTonicClonicSeizure bits (including subtypes)
Only myoclonic tonic-clonic seizures	Classification	(s & MyoclonicTonicClonicSeizure) === MyoclonicTonicClonicSeizure	Labels containing all MyoclonicTonicClonicSeizure bits (including subtypes)
Events with a tonic component	Combination	(s & Tonic) === Tonic	Labels containing a Tonic bit or descriptor
Events with an elementary motor phenomenon	Combination	s & (ElementaryMotorTraitMask | ElementaryMotorPhenomenon)	Labels containing an elementary motor trait bit or descriptor
Events with a complex motor phenomenon	Descriptors	(s & ComplexMotorPhenomenon) === ComplexMotorPhenomenon	Labels containing a descriptor in the complex motor phenomenon list
Events with any motor phenomenon	Combination	s & (ElementaryMotorTraitMask | MotorPhenomenonMask)	Labels containing a bit or descriptor in with any motor bit
Seizures with postictal phenomena	Descriptors	(s & Postictal) === Postictal	Labels with at least one postictal descriptor
Events with a myoclonic component	Combination	(s & Myoclonic) === Myoclonic	Labels containing a Myoclonic bit or descriptor
Events with an absence component	Combination	(s & Absence) === Absence	Labels containing the Absence bit or descriptor
Events with an atypical component	Combination	(s & Atypical) === Atypical	Labels containing the Atypical bit or descriptor
Events with an negative myoclonic component	Combination	(s & NegativeMyoclonic) === NegativeMyoclonic	Labels containing the NegativeMyoclonic bits
Events with an atypical absence component	Combination	(s & AtypicalAbsence) === AtypicalAbsence	Labels containing the AtypicalAbsence bits
Only typical absence seizures	Classification	s === TypicalAbsenceSeizure	Labels where the canonical seizure type is exclusively TypicalAbsenceSeizure
Only atypical absence seizures	Classification	s === AtypicalAbsenceSeizure	Labels where the canonical seizure type is exclusively AtypicalAbsenceSeizure

While every clinical aspect is intended to have its own bit, it is worth reiterating that some aspects are “implicit” or “default” in the proposed model when a range of relevant values is unset. In other words, the complete absence of a set of related or mutually exclusive traits may imply a logically separate trait that has been assigned a zero value. Such values (i.e., UnknownOrigin, UnknownConsciousness, Symmetric) require special attention in order to achieve semantically-correct results when using them as comparators, as a simple ANDing will always be falsy (return zero). While the proposed encoding defines only a few zero-value identifiers, these must be handled with care when used as a comparator: the operation should always compare the masked (set of known search values) to the target “default” (zero-valued) trait. To this end, example operations listed in Table 6 use both a mask and a comparator (where applicable) in order to generalize against this special constraint (even in cases where a simple truthiness check on the mask would be enough).

As the table shows, a wide range of potential use cases can be encoded in a framework using bitwise operations on the proposed structured encoding. Notably, the bottom seven rows specifically deal with the potential ambiguity of the Atypical bit. In every case but the “events with an atypical component” (the only search that considers only the Atypical bit), no “unintended” results are expected. In that case, though, both negative myoclonic and atypical absence seizures would be returned, which might not be a clinically useful search case. Any single-bit trait query (e.g., Tonic or Clonic bits returning both Focal and Generalized seizure types) may cut across sections of search values. Implementers must be aware of this fact, be aware of the semantics, and use a sufficient number of bits to distinguish between groups. It would not be appropriate, for example, to train a machine learning model based on labels derived from single trait bits; seizure subtypes or fully-specified canonical types would provide greater entropy for labeling purposes.

### 3.8 Mapping expanded seizure classifications using the proposed structure

To evaluate the effectiveness of the model to produce accurate expanded structure, a subset of the more complex vignettes were examined from the practical guide. Each semiology has been numerically modeled accordingly.

#### 3.8.1 Example 1

From the practical guide's clinical vignettes, case 20:

A 12-year-old boy presents with his first seizure[…] he initially rotates his body to the right, followed by a forced head version to the right and eye deviation to the left. He then falls to the ground, exhibiting bilateral stiffening of all limbs and clonic jerks that gradually decrease in frequency toward the end of the episode. This seizure is classified as a **focal-to-bilateral tonic-clonic seizure**, with the following semiology: **gyratory movement to the right** → **versive movement to the right + eye deviation to the left** → **bilateral tonic-clonic**.

The resulting classification table is:

**Table:** 

Seizure classification		Hexadecimal
FocalToBilateralTonicClonicSeizure		0x0000000000000000 0000000000000033
**Descriptor**	**Rank**	
Gyratory | Right	1	0x0000000000000000 0000004001008000
Versive | Right	2	0x0000000000000000 0000040001008000
EyeDeviation | Left	2	0x0000000000000000 0000002001004000
TonicClonic | Bilateral	3	0x0000000000000000 000000000100C020
**Combined value**		0x0000000000000000 000004600100C033

Note that the versive movement and eye deviation share the same rank (2), allowing an application to visualize them using the + operator. Allowing a distinction between index (the raw order) and rank allows for the natural order to be retained while allowing for transitions between simultaneously-grouped phenomena.

#### 3.8.2 Example 2

From the practical guide's clinical vignettes, case 22:

A 28-year-old right-handed man has presented with recurrent extension spasms since age 3 […] seizures changed at age 12, beginning with a feeling of depersonalization […] followed by chest tightness and facial flushing […]. He was unable to respond verbally to calls, continuing with hyperkinetic movements (punching and kicking) and screaming for less than a minute. At the end, he reported recalling what happened […] [t]he patient's unresponsiveness was due to ictal aphasia and the ongoing hyperkinetic movement, not impaired consciousness, as evidenced by his ability to respond to nonverbal cues and his intact memory. This seizure is classified as a **focal preserved consciousness seizure**, with the following semiology: **depersonalization aura** → **flushing** → **hyperkinetic complex automatisms + aphasia** → **postictal nose wiping with the left hand**.

The full classification is thus:

**Table:** 

Seizure classification		Hexadecimal
FocalPreservedConsciousnessSeizure		0x000000000000000 0000000000000000B
**Descriptor**	**Rank**	
Depersonalization	1	0x0000000000000000 0200000004000000
Flushing	2	0x000000000000C000 0000000010000000
Hyperkinetic	3	0x0000000000000000 0020000002000000
VocalAutomatisms	3	0x0000000000000000 0010100002000000
Aphasia	3	0x0000000000000004 0000000008000000
NoseWiping	4	0x0082000000000000 0000000000000000
**Combined value**		0x008200000000C004 023010001E00000B

All phenomena listed in the seizure description exist in the canonical list. While it was not in the original classification, the text also lists screaming, so VocalAutomatisms was added to rank 3.

#### 3.8.3 Example 3

From the practical guide's clinical vignettes, case 29:

A 22-year-old man developed seizures at 17 years of age […]. Focal seizures were described as typically occurring in prolonged clusters, intermittently evolving to left head deviation prior to bilateral convulsions and status epilepticus […]. These [two seizure types] are classified as 1) **focal preserved consciousness seizure**, with the following semiology: **aura (flushed, strange feeling or déjà vu) + preserved responsiveness and awareness**, and 2) **focal-to-bilateral tonic-clonic seizure**, with semiology **starting as type #1** and then evolving to **[versive] head deviation to the left and ([**→**]) bilateral tonic-clonic** phase.

This patient has focal seizures that sometimes transition into focal-to-bilateral tonic-clonic seizures (in the pre-2017 terminology, this would be known as a “secondary generalization”). The first type is defined as:

**Table:** 

Seizure classification		Hexadecimal
FocalPreservedConsciousnessSeizure		0x0000000000000000 000000000000000B
**Descriptor**	**Rank**	
IndescribableAura	1	0x0000000000000000 0000000040000000
Flushing	1	0x000000000000C000 0000000010000000
DejaVu	1	0x0000000000000040 0000000008000000
PreservedConsciousness	1	0x0000000000000000 0000000000000008
**Combined value**		0x000000000000C040 000000005800000B

Notably, the vignette mentions “preserved responsiveness and awareness” explicitly as a descriptor, despite having that trait from the basic classification. Also, we note that the original used the term deviation; this is most likely intended to mean versive head movement. This is useful to retain as a descriptor when considering the “evolved” form of the seizure, as it allows modeling of the preserved consciousness state prior to the tonic-clonic phase:

**Table:** 

Seizure classification		Hexadecimal
FocalToBilateralTonicClonicSeizure		0x0000000000000000 0000000000000033
**Descriptor**	**Rank**	
IndescribableAura	1	0x0000000000000000 0000000040000000
Flushing	1	0x000000000000C000 0000000010000000
DejaVu	1	0x0000000000000040 0000000008000000
PreservedConsciousness	1	0x0000000000000000 0000000000000008
Versive | Head | Left	2	0x0000000000000000 0000040001104000
TonicClonic | Bilateral	3	0x0000000000000000 000000000100C020
**Combined value**		0x000000000000C040 000004005910C03B

#### 3.8.4 Example 4

From the practical guide's clinical vignettes, case 16:

An 18-year-old woman with moderate intellectual disability had onset of epilepsy at age three years, and her seizures have been drug-resistant […] more recently she has had increasing episodes […]. These are not followed by clonic activity. […] [h]er seizure begins abruptly with generalized body stiffening and upward eye rolling, and her EEG clearly shows a bilateral-synchronous/generalized onset. Although her eyes can deviate slightly to the left, this is still considered a generalized seizure. This seizure is classified as a **generalized tonic seizure**.

Strictly following the position paper, there is no need to classify the seizure beyond its type of GeneralizedTonicSeizure. However, this is an interesting case involving semiological features that may have led to a different diagnosis depending on the available evidence. Such a classification might be defined as follows:

**Table:** 

Seizure classification		Hexadecimal
GeneralizedTonicSeizure		0x0000000000000000 0000000000001005
**Descriptor**	**Rank**	
EyeDeviation | Left Tonic | Bilateral	1 1	0x0000000000000000 0000002001004000 0x0000000000000000 000000000100D000
**Combined value**		0x0000000000000000 000000200100D005

#### 3.8.5 Example 5

The position paper discusses various tonic seizure subtypes, such as those in CDKL5 deficiency disorder, that are the subject of emerging fields of research. A recent case study (Appendino, 2022) provides such a description:

[A] nine-month-old female [presents] with a typical hypermotor-tonic-spasms seizure sequence associated with CDKL5 deficiency disorder. From a drowsy-quiet state at the onset of the video, the patient suddenly becomes agitated, exhibiting a very brief hypermotor phase with both hips flexed alternatingly. A tonic phase involving all four limbs occurs simultaneously with bilateral anterior quadrant attenuation on the EEG. This lasts for about 12 seconds, followed by the spasm phase, which lasts for almost five minutes[…]. The patient cries after each spasm in the first half of the cluster.

As the position paper frames it, “hypermotor-tonic-spasms” is a colloquial term derived from semiology and is not a classifier alone. The case study describes the seizure as generalized and it fits no existing syndromic subtype, so a valid interpretation would likely be **generalized seizure** with semiology **hyperkinetic behavior** → **tonic bilateral-symmetric** → **epileptic spasms (cluster)**. It is noted that the spasms occur in a cluster, and the patient cries between spasms. Therefore, the crying is not considered an ictal activity, but the cluster nature of the seizure should be noted in the semiology. Particularly because this seizure type represents a generalized seizure not incorporated as a canonical subtype, it serves as another example where a generalized seizure may indeed benefit from further physiological details in the classification.

**Table:** 

Seizure classification		Hexadecimal
GeneralizedSeizure		0x0000000000000000 0000000000000005
**Descriptor**	**Rank**	
Hyperkinetic Tonic | BilateralSymmetric Spasm with free text “cluster”	1 2 3	0x0000000000000000 0020000002000000 0x0000000000000000 000000000100D000 0x0000000000000000 0000000001000800
**Combined value**		0x0000000000000000 002000000300D805

As alluded to in the case description, the semiology is “typical”, suggesting that a separate descriptor might provide a better description of the phenomenon (similarly to how the “tonic-clonic” descriptor is separate from the “tonic” and “clonic” descriptors). Such a descriptor could be defined combining relevant bits and free text as follows:

**Table:** 

Seizure classification		Hexadecimal
GeneralizedSeizure		0x0000000000000000 0000000000000005
**Descriptor**	**Rank**	
Hyperkinetic | Tonic | BilateralSymmetric | Spasm with free text “hyperkinetic-tonic-spasms cluster”	1	0x0000000000000000 002000000300D800
**Combined value**		0x0000000000000000 002000000300D805

As the descriptor was defined to use the combined value of the four descriptors used above, the two approaches yield the same numeric identity. While these two models may be effectively equivalent for many use cases (such as searches for events containing these traits), they indicate an inherent trade-off between the granularity of temporal subevents vs. specific labeling of a “well-known” complex motor sequence. This choice may, for instance, affect the training outcomes when building machine learning models from these labels.

### 3.9 An end-to-end example

The repository contains an example web application that demonstrates the proposed bitwise encoding in an end-to-end software environment. The client side is a single-page web application (SPA) written in vanilla JavaScript (transpiled from TypeScript) using the definitions within the library. The server side is an HTTP API, written in Rust using the Rocket (Benitez, 2026) framework, and PostgreSQL as the storage solution. Features of the example include:

- Querying for all “events” (using the simple data model described above) using bitwise searches (loose, strict, and zero-mask searches are supported).
- Creating new events from a hexadecimal value and optional free text
- Displaying the canonical type matching and validation information via the validate() function

Figure 3 provides a screenshot of the web client. Filtering is performed on the backend, using PostgreSQL's bitwise arithmetic support for bit string fields. As Rocket provides an object-relational mapping (ORM) programming model, the Event interface is mapped to and from JSON within the endpoint definitions with minimal boilerplate code. However, custom serialization/deserialization functions were required to allow for hexadecimal strings to transit seamlessly between the incoming, outgoing, and internal (SQL) APIs. Notably, the following elements were required:

- When serializing a u128 value for the outgoing API, the default serializer was overridden to write a 0x-prefixed hexadecimal string.
- When deserializing an incoming hexadecimal string, a custom deserializer was used to construct a corresponding u128 integer (using the expression noted in Table 2).
- When performing queries against the database, u128 values were converted to bit vectors (BitVec) via the standard library's support for viewing integers as a byte slice.

**Figure 3:**
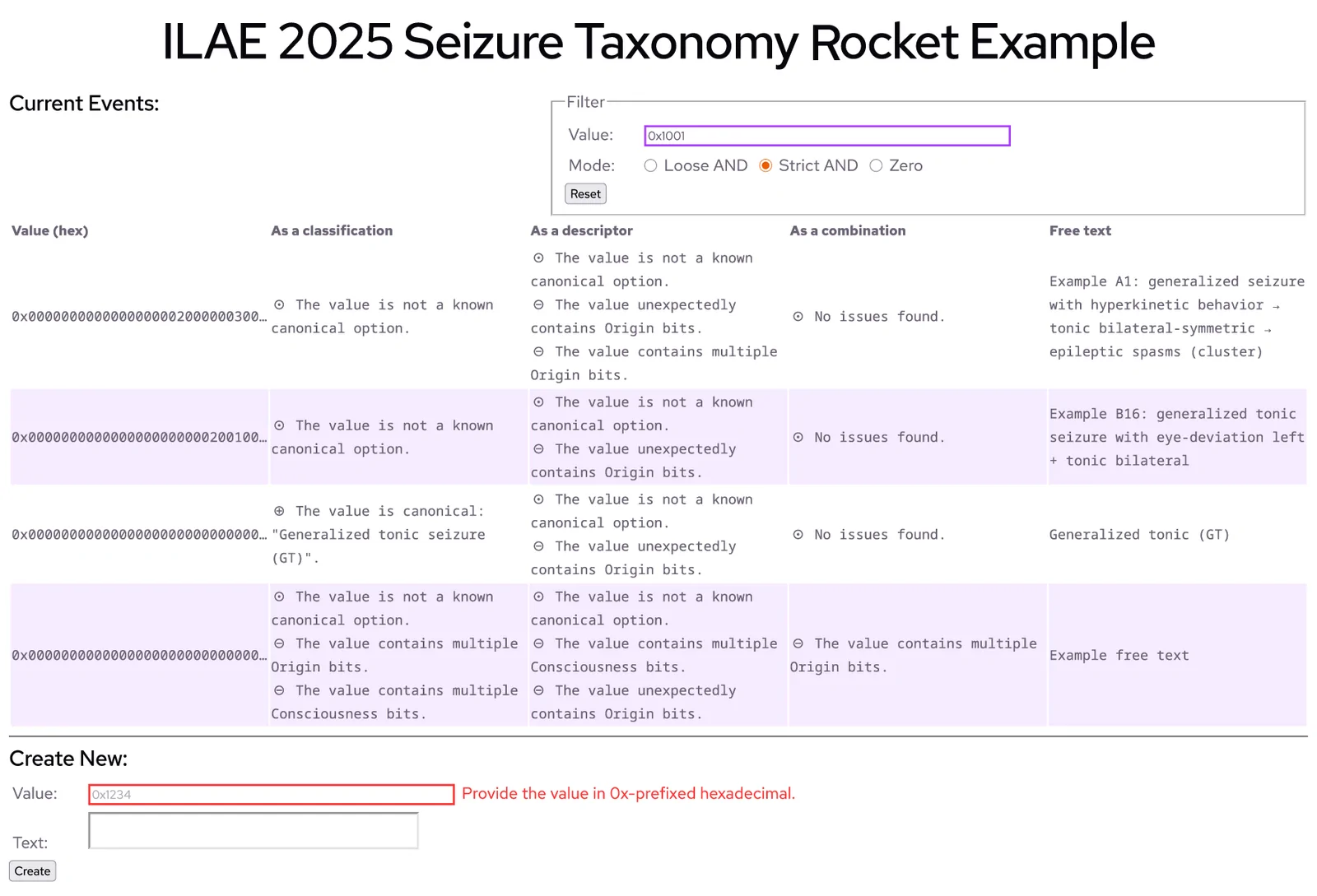
The repository contains an example web user interface interacting with a Rust-based backend. The user can create and query values using the encoding system defined by the library.

As a consequence, values transition between JavaScript BigIntegers, JSON strings, Rust u128 bit integers, Rust BitVec, and PostgreSQL bit(128) types—and back—within each client-server interaction. While the representation changes across these transitions, the underlying values stay the same, demonstrating that this approach is applicable to the types of programming environments encountered in modern medical software.

## 4 Discussion

### 4.1 Limitations

As there are trade-offs with any design choices dealing with limited resources (such as the constraints on the number of available bits), some limitations have been noted. While many examples have been provided, the proposal may still have “blind spots” for certain types of bitwise operations. The implementer must understand the semantics of these operations and choose wisely; this complexity may lead to cases where clinically conflicting values are chosen. As noted in Table 3, while the validate() method has been provided, there may be application-specific cases where antipatterns can be detected and highlighted to the user. For example, it is clinically unlikely that a TonicClonic descriptor would be attached to a seizure that is not tonic-clonic (although it may certainly be done during the course of the classification process). Using a set of predefined rules, the user interface could highlight such discrepancies and guide the user to select a more “clinically understood” combination, even when the resulting bit pattern would be technically valid.

The “economical” principle behind the proposal has led it to use all available 32-bit values, potentially making it inflexible for directly extending or modifying in the future. While this is mitigated by specification of Other bit (allowing any extensions to extend this value), it implies that the rest of the bits might be reused for other purposes, increasing the risk of bugs or requiring extra filtering within the implementation. A similar problem arises when considering the Atypical trait bit, as it is used in two different subtypes of generalized seizures, potentially leading to unintended search queries when precise search semantics are not used. One potential design change would be to move the somatotopic modifiers entirely into the DescriptorType structure, freeing up bits in the base integer for other uses. A further refactoring could see moving all the masks (classifiers, somatotopic modifiers, descriptor groups, etc.) into their own integer bitmaps, to recombine when needed using big integer shifts. While this would require multiple integer bitmaps, it would open up additional bits within each group for greater flexibility when extending the model. While consistent with how descriptors have already been proposed to work, it has the disadvantage of introducing more complexity before strictly required.

### 4.2 Future work

It is important to recognize that this proposal has been scoped to be a model for binary representation of the ILAE 2025 system. It does not offer a complete implementation or integration with a specific framework. No specific solution is proposed for use in relational database systems, for example; it is up to the implementers to decide how to integrate the proposal into their software. Future work which would be fitting for the code repository might include:

- Providing additional conformance tests and reference implementations in other popular programming and database languages
- Adding additional test cases (like the five examples in the previous section) showing how the structured classification text (i.e., not only the numeric value) can be mapped into the proposed numeric model
- Defining benchmarks to measure how, when used with a prompt explaining the programming model, an LLM can be used to transform unstructured text into the standardized classification text and/or the numeric model
- Defining benchmarks to test the performance of the bitwise search mechanism under various scenarios and database layouts, comparing between different column types such as integer, bitfield, and vector
- Exploring the possibility to infer lateralization and/or localization signs based on the chosen combination of classifiers and descriptors
- Defining mappings to established health interoperability systems and databases such as FHIR, SNOMED CT (SNOMED International, 2026), the Observational Medical Outcomes Partnership Common Data Model (OMOP CDM) (Observational Health Data Sciences and Informatics, 2026), and the Open Biological and Biomedical Ontology (OBO) Epilepsy Ontology (EPIO) (Sargsyan et al., 2023).

Regarding the last item, a preliminary mapping has been considered in Table 7.

**Table 7:** Possible mappings to common computer-processable clinical documentation systems.

System	Mapping
HL7 FHIR	A custom Coding would allow for storing complete classification information as a CodeableConcept within an Observation. For simpler applications, a hexadecimal string encoding could be placed in Observation.valueString.
SNOMED CT	A Refset for existing SNOMED CT epilepsy disorders to ILAE 2025 canonical/encoded types would provide a mapping to the myriad epileptic disorder concepts defined within the catalog.
OMOP CDM	A Concept and associated Vocabulary would model the ILAE 2025 domain, allowing for Observations with values encoded as concept IDs or hexadecimal strings.
OBO EPIO	A new ILAE 2025 OBO library could be defined, allowing for encoded types to be referenced in EPIO through an owl:annotatedSource tag on existing seizure type definitions.

As our work continues and feedback from clinicians and the wider community is received, we anticipate that many of these issues will be addressed within the code repository. More comprehensive data exchange mechanisms, such as those noted in Table 7, will require wider collaboration with the respective interoperability communities.

### 4.3 Governance and versioning

At the time of this article's writing, the linked software repository is in its early exploratory stages, and has not been integrated into any commercial products. It is therefore likely to evolve over time, based on both the experience of the authors and via community feedback. Maintainership (in terms of merging code modifications, testing, and releasing) is therefore provided by the authors, with the possibility of including other contributors or a wider consortium of users in the future. This company-backed founder-leader model (Red Hat Inc., 2021) is common among small open-source projects (including those in the ontology space, such as the aforementioned EPIO). Should the project attract active stakeholders and users, the authors are open to transitioning to a consortium-style model (with a self-appointing council or board) to better serve the needs of the community. Feedback may currently be provided via the project site's issue mechanism or by contacting the authors.

Versioning has been designated to follow Semantic Versioning 2.0.0 (Preston-Werner and The SemVer Team, 2013), providing guidelines for how code changes may be introduced over time. Once the project has reached version 1.0, backwards-incompatible changes (such as modifications to numeric encodings) may not be made without a major release. Implementers will be expected to migrate any encodings they have stored—such as those within SQL databases—when upgrading to a major version of the library/specification. While the project has already outlined a policy to document impact assessments as part of its changelog, it may offer migration helper scripts in the future as needed.

Similarly, the ILAE working group is likely to adjust its own guidance in the future; depending on the scope and impact of such future changes, this project may receive updates to adhere to the updated standard. On a practical level, such updates must follow the existing principles of backwards compatibility and numeric migration; that is, implementers should have a clear path to upgrading the library, and migrate their own stored values, without data loss or large refactoring effort. If more dramatic shifts in how the ILAE views seizure classification should occur, an entirely new project may be viewed as a better approach than attempting to juggle multiple disparate seizure classification schemes within one library.

### 4.4 Conclusion

The ILAE 2025 position paper and associated practical guide have established an updated standard to which both healthcare providers and medical device software providers working with epilepsy patients are expected to adhere. By outlining a precise and compact numerical strategy for documenting seizure types under the new standard, the information within the type system can be faithfully captured and stored within information systems. Through the use of bitwise arithmetic, many useful properties can be encoded into the type system, allowing for efficient and intuitive search within seizure databases. Such searches can ostensibly aid in selecting samples for machine learning model development, as well as aiding clinicians in finding good examples of documented seizures for training and triage. These rules can also help migrate existing datasets to the new standard, aligning legacy data with incoming data.

As noted in the examples, a clinical workflow is likely to produce descriptors for seizures of all types. While descriptors are not used in the formal classification of generalized seizures, we identify clinical benefits of being able to record and retain them even when the final classification is a generalized seizure type. Like the introduction of new descriptors or non-epileptic indications, we note that medical databases are likely required to offer more flexibility in the underlying storage than strictly what is proposed by the 2025 standard. Since epileptic seizures are complex and diverse—manifesting with epileptic symptoms of variable clinical expression, appearing either as the sole type or in combination—free use of descriptors is mandatory to meet the expectations of clinical personnel.

Conversely, injudicious use of novel descriptors may be considered reckless, particularly if the information system is not designed to collate identical or similar descriptors into the same underlying type. Certainly, systems incorporating tools such as LLMs can leverage value from less structured data, but this still runs counter to the core tenet of this proposal: to keep the system as structured and compact as possible. Use of predefined descriptors is therefore desirable, but the implementers should carefully consider how to allow users to expand the available descriptor list in a way that retains the orthogonal semiological concepts defined by the ILAE 2025 type system.

While the proposed framework has some observed limitations and potential extensibility constraints, it is a shared starting point for software systems to update to the new standard. As of version 0.0.4, the implementation is exploratory and should not be considered a final design. Its intent to serve as an open reference specification for system implementers is in the spirit of interoperability and compatibility, and the authors intend to maintain and update the specification based on their own experience and via input from the community. Through promotion of a common implementation and set of recommended software approaches, awareness and adoption of the ILAE 2025 standard can be raised, ultimately improving patient care through more precise clinical documentation.

## Data Availability

The datasets presented in this study can be found in online repositories. The names of the repository/repositories and accession number(s) can be found below: https://gitlab.com/neuroeventlabs/public/ilae2025.
